# A novel soluble complement receptor 1 fragment with enhanced therapeutic potential

**DOI:** 10.1074/jbc.RA120.016127

**Published:** 2020-12-23

**Authors:** Sandra Wymann, Yun Dai, Anup G. Nair, Helen Cao, Glenn A. Powers, Anna Schnell, Genevieve Martin-Roussety, David Leong, Jason Simmonds, Kim G. Lieu, Mitchell J. de Souza, Marcel Mischnik, Shirley Taylor, Saw Yen Ow, Martin Spycher, Rebecca E. Butcher, Martin Pearse, Adrian W. Zuercher, Adriana Baz Morelli, Con Panousis, Michael J. Wilson, Tony Rowe, Matthew P. Hardy

**Affiliations:** 1 Research and Development, CSL Behring AG, Bern, Switzerland; 2 CSL Ltd, Bio21 Institute, Victoria, Australia; 3 Research and Development, CSL Behring GmbH, Marburg, Germany

**Keywords:** CSL040, complement, receptor, recombinant protein expression, C3b, pharmacokinetics, glycosylation, glomerulonephritis, 2-AB, 2-aminobenzamide, AP, alternative pathway, AUC, area under the curve, CP, classical pathway, CR1, complement receptor 1, DHAP, 2,5-dihydroxyacetophenone, GBM, glomerular basement membrane, GN, glomerulonephritis, GVB, gelatin veronal buffer, HRP, horseradish peroxidase, HuCR1, human complement receptor 1, IgG, immunoglobulin G, LHR, long homologous repeat, MALS, multiangle light scattering, MRT, mean residence time, MsαRb, mouse anti-rabbit monoclonal IgG, MW, molecular weight, NHS, normal human serum, NTA, nitrilotriacetic acid, PNGase, peptide:N-glycosidase, SCR, short consensus repeat, SEC, size-exclusion chromatography, SPR, surface plasmon resonance, TMB, 3,3′5,5′-tetramethylbenzidine

## Abstract

Human complement receptor 1 (HuCR1) is a pivotal regulator of complement activity, acting on all three complement pathways as a membrane-bound receptor of C3b/C4b, C3/C5 convertase decay accelerator, and cofactor for factor I-mediated cleavage of C3b and C4b. In this study, we sought to identify a minimal soluble fragment of HuCR1, which retains the complement regulatory activity of the wildtype protein. To this end, we generated recombinant, soluble, and truncated versions of HuCR1 and compared their ability to inhibit complement activation *in vitro* using multiple assays. A soluble form of HuCR1, truncated at amino acid 1392 and designated CSL040, was found to be a more potent inhibitor than all other truncation variants tested. CSL040 retained its affinity to both C3b and C4b as well as its cleavage and decay acceleration activity and was found to be stable under a range of buffer conditions. Pharmacokinetic studies in mice demonstrated that the level of sialylation is a major determinant of CSL040 clearance *in vivo*. CSL040 also showed an improved pharmacokinetic profile compared with the full extracellular domain of HuCR1. The *in vivo* effects of CSL040 on acute complement-mediated kidney damage were tested in an attenuated passive antiglomerular basement membrane antibody-induced glomerulonephritis model. In this model, CSL040 at 20 and 60 mg/kg significantly attenuated kidney damage at 24 h, with significant reductions in cellular infiltrates and urine albumin, consistent with protection from kidney damage. CSL040 thus represents a potential therapeutic candidate for the treatment of complement-mediated disorders.

The complement system, comprising more than 30 proteins in plasma and on cell surfaces, is a key element of the innate immune system and a primary mechanism of host defense against pathogens. It also functions as an important link to the adaptive immune and coagulation systems (1, 2, 3). Targeting of cells and immune complexes by complement results in phagocytosis *via* C4b/C3b-mediated opsonization, direct cell lysis through the formation of the pore-forming membrane attack complex, and in inflammation mediated primarily through the anaphylatoxins C3a and C5a. The complement cascade is initiated *via* the classical pathway (CP), lectin pathway, and alternative pathway (AP) by a variety of activators ranging from immune complexes, neoepitopes, endotoxin, certain carbohydrates, and in the case of the AP, spontaneously by what is referred to as a tick-over mechanism (4) involving hydrolysis of C3. These pathways converge at the level of C3, leading to cellular activation, phagocytosis, or lysis (1). Regulatory proteins, including complement receptor 1 (CR1) (5), factor H (6), factor I (7), CD46 (8), CD55 (9), and CD59 (10), have evolved to tightly regulate the complement cascade and prevent unintended damage to host cells or tissues.

In addition to its role in pathogen clearance, complement is intimately involved in the pathogenesis of a number of human disease states, such as post-transplant graft rejection and ischemia reperfusion injury (11, 12, 13, 14, 15), atypical hemolytic uremic syndrome (16), glomerulonephritis (GN) (17, 18), paroxysmal nocturnal hemoglobinuria (19), myasthenia gravis (20), systemic lupus erythematosus (21), macular degeneration (22), and septic shock (23). These diseases and/or injuries can occur not only when complement is hyperactivated but also when expression of fluid-phase or membrane-associated complement regulation is aberrant (1, 24, 25). Mutations in genes that encode complement proteins are also associated with disease (26). Examples of this are the association of the factor H gene mutations with atypical hemolytic uremic syndrome (27), C1q gene mutations with a systemic lupus erythematosus–like syndrome (28), and C9 gene mutations with meningococcal infection (29).

Human complement receptor 1 (HuCR1), also known as CD35, is type I membrane protein that forms part of a family of complement receptors that include CR2, CR3, and CR4 (5). It is expressed on erythrocytes, eosinophils, monocytes, macrophages, neutrophils, B and some T cells, mast cells, glomerular podocytes, and follicular dendritic cells (30). The extracellular domain of HuCR1 is 1971 amino acids long and composed of 30 short consensus repeat (SCR) domains, each of approximately 60 amino acids and of variable homology to each other (31, 32). SCR domains 1 to 28 of HuCR1 are further arranged into four long homologous repeat (LHR) domains, each consisting of seven SCR domains (31, 33). Very low levels (approximately 50 ng/ml) of soluble HuCR1 occur naturally in plasma, likely produced as a consequence of proteolytic shedding (34).

HuCR1 has multiple functions. It acts as the receptor for both C3b and C4b, and to a lesser extent, iC3b and C3dg (35, 36, 37). It also acts as a cofactor for factor I-mediated cleavage of C3b to iC3b, and for further cleavage of iC3b to C3dg and C3c (38, 39). A similar cofactor function for C4b cleavage has also been observed (40). The third main function of HuCR1 is to regulate complement by accelerating convertase decay *via* binding to C3b or C4b and displacing C2a or Bb in the C3 or C5 convertases (36, 40). Additional functions, such as clearance of immune complexes (41) and enhancement of phagocytosis (42), have also been described. HuCR1 inhibits complement activity in multiple species, including mouse (43, 44, 45), rat (46, 47, 48, 49, 50), rabbit (51, 52), and pig (53, 54).

A recombinant, soluble, and full extracellular domain form of HuCR1 comprising of all 28 SCR domains and known as TP-10 has previously been developed for therapeutic use (55). Phase I open-label ascending dose study (56) showed that TP-10 had a mean half-life of 69 h, with doses greater than 1 mg/kg inhibiting complement activity. Two clinical trials examining the effect of TP-10–mediated complement inhibition in patients during cardiopulmonary bypass demonstrated a gender-specific effect, with only males showing clinically significant improvement (57, 58). However, a study testing TP-10 at 10 mg/kg in 59 lung transplant recipients (59) has shown clinical efficacy, with 50% of patients in the TP-10 group extubated at 24 h compared with 19% in the placebo group. In addition, the mean duration of mechanical ventilation was reduced to 10.6 days in TP-10–treated patients compared with 21.5 days in standard-of-care patients (59). This clinical trial data show that not only that a soluble CR1 has potential value as a therapeutic but also the choice of indication is important.

In this report, we assessed the role of individual and combined HuCR1 LHR domains by comparing their complement inhibitory activity as soluble proteins in both ELISA-based and red blood cell hemolytic assays. From these *in vitro* studies, we identified and characterized CSL040, a truncated soluble variant of HuCR1 lacking the LHR-D domain exhibiting greater *in vitro* potency and an extended *in vivo* half-life compared with full-length HuCR1. We demonstrated the importance of sialylation to reduce *in vivo* clearance rate of CSL040 as well as its ability to attenuate damage *in vivo* in a mouse model of immune complex–mediated kidney injury.

## Results

### Identification of CSL040, a soluble truncation variant of HuCR1

**Figure 1:**
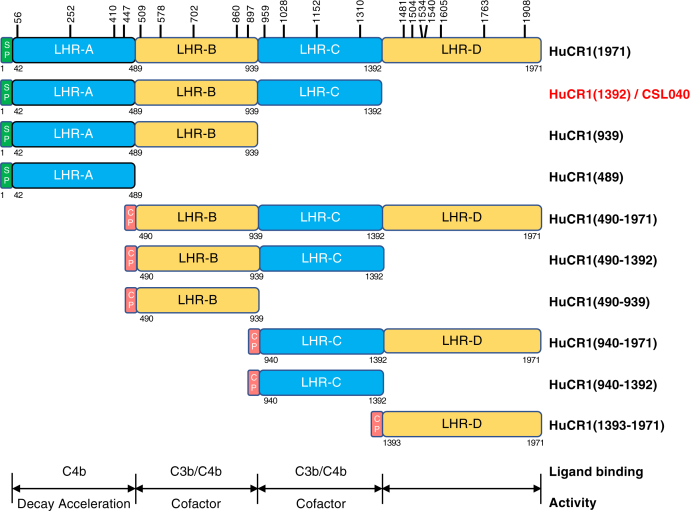
**Schematic representation of N- and C-terminal soluble recombinant truncation mutants of HuCR1.** LHR domain boundaries are based on (27) and from UniProt P17927 (https://www.uniprot.org). The *green boxes* denoted SP indicate the endogenous signal peptide, and *red boxes* denoted CP indicate the 19aa signal peptide from ceruloplasmin (GenBank accession number: NP_000087). Vertical bars with numbers above HuCR1(1971) denote the amino acid position of N-glycosylation sites. Amino acid numbering is based on Met + 1. Numbers below each construct indicate the amino acid position of the C-terminal end of the LHR domain boundaries, with the exception being at the N-terminal end of the mature sequence where the numbering denotes the start of the respective domain. Underneath the schematic is shown the known LHR domain(s) within CR1 responsible for both C3b/C4b ligand binding, decay acceleration activity, and cofactor activity, based on (5). HuCR1, human complement receptor 1; LHR, long homologous repeat.

We sought to identify potential therapeutic candidates based on HuCR1 by determining the relative contributions of the LHR domains of soluble HuCR1 to its overall potency *in vitro*. A series of recombinant soluble N-terminal and C-terminal truncation mutants were generated that lacked 1, 2, or 3 of its four LHR domains (Fig. 1) and were shown to be monomeric by SDS–PAGE (Fig. S1). These proteins were purified, and their potencies compared with the full-length extracellular domain HuCR1(1971) *in vitro*. Using the Wieslab complement system kits for all three complement pathways or red blood cell hemolytic assays specific for the CP and AP (Table 1), we showed that purified single LHR domain variants of HuCR1 had greatly reduced complement inhibitory activity *in vitro* compared with HuCR1(1971). Indeed, recombinant HuCR1(1393–1971) had no detectable activity (Table 1) in any of the assays employed. Mutants with three contiguous LHR domains (LHR-AB, LHR-BC, and LHR-CD) exhibited increased inhibitory activity compared with singly expressed LHR domains, with HuCR1(939) generally being more potent than HuCR1(490–1392) and HuCR1(940–1971) (Table 1). HuCR1(490–1971) had lower activity than soluble full-length HuCR1(1971), particularly as measured in the hemolytic assays; although for some assays, this was not determined to be statistically significant (Table 1). Surprisingly, HuCR1(1392) containing LHR-ABC was a significantly more potent inhibitor than full-length HuCR1(1971), of two of three pathways in the Wieslab assay, and both hemolytic assays (Table 1). The potency improvements for HuCR1(1392) over HuCR1(1971) were found to be approximately twofold for both the CP and lectin pathway–specific assays and approximately threefold for both Wieslab and hemolytic AP assays (Table 1). Removal of the histidine (His)-tag from HuCR1(1392) had no effect on its potency *in vitro* (Fig. S2). HuCR1(1392) was designated as CSL040 and is described as such in the remainder of the article.

**Table 1:** Potency of human soluble CR1 truncation variants in complement pathway–specific Wieslab and red blood cell hemolytic assays

HuCR1 truncations		Wieslab IC_50_ (nM) ± SEM			Hemolytic IC_50_ (nM) ± SEM	
Construct	LHR domains	Classical	Lectin	Alternative	Classical	Alternative
HuCR1(1971)	ABCD	1.28 ± 0.26	1.05 ± 0.17	0.53 ± 0.29	1.21 ± 0.21	2.71 ± 0.94
HuCR1(1392)/CSL040	ABC	0.53 ± 0.33^a^	0.47 ± 0.16^a^	0.17 ± 0.10	0.42 ± 0.10^b^	0.90 ± 0.54^a^
HuCR1(490–1971)	BCD	4.18 ± 2.49	3.12 ± 0.59^b^	0.29 ± 0.17	32.96 ± 15.34^a^	76.85 ± 49.15
HuCR1(939)	AB	2.59 ± 1.30	1.87 ± 0.64	1.31 ± 1.82	4.61 ± 1.85^a^	13.15 ± 2.18^b^
HuCR1(490–1392)	BC	10.06 ± 2.55^b^	5.80 ± 0.65^c^	0.70 ± 0.36	10.08 ± 5.52^a^	12.48 ± 4.30^a^
HuCR1(940–1971)	CD	98.57 ± 71.68	120.74 ± 49.29^a^	1.26 ± 1.17	677.80 ± 278.35^a^	95.51 ± 21.06^b^
HuCR1(489)	A	36.91 ± 7.25^b^	22.35 ± 14.36	1.10 ± 1.43	68.38 ± 9.52^c^	49.87 ± 24.76^a^
HuCR1(490–939)	B	No activity	No activity	8.18 ± 9.63	959.37 ± 243.92^b^	63.38 ± 31.03^a^
HuCR1(940–1392)	C	No activity	1879.7 ± 1545.3	0.79 ± 0.61	733.10 ± 203.47^b^	66.36 ± 18.10^b^
HuCR1(1393–1971)	D	No activity	No activity	No activity	No activity	No activity

The IC_50_ values listed are the mean ± SD of three independent experiments. Statistically significant differences for individual HuCR1 fragment IC_50_ values compared with parental HuCR1(1971) for each pathway assay were calculated by one-way ANOVA.

CR1, complement receptor 1; HuCR1, human complement receptor 1; LHR, long homologous repeat.

a *p* < 0.05.

b *p* < 0.005.

c *p* < 0.0005.

### Biochemical analysis of CSL040

**Figure 2:**
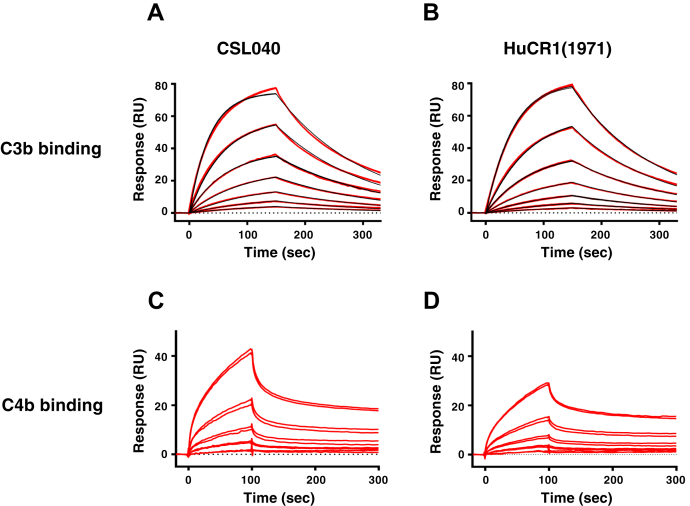
**Comparative affinity of CSL040 and CR1(1971) to human C3b and C4b.** Biosensor data of plasma-derived human C3b binding to (*A*) CSL040 and (*B*) HuCR1(1971). Panels show double-referenced sensorgrams from a series of seven analyte concentrations (15.6, 31.3, 62.5, 125, 250, 500, and 1000 nM), injected for 150 s with dissociation monitored for 180 s. Each *red line* represents two overlaid sensorgrams. All C3b data were fit to a kinetic 1:1 model (*black lines*) including a term for mass transport. Calculated kinetic data are shown in Table 2. Biosensor data of plasma-derived human C4b binding to (*C*) CSL040 and (*D*) HuCR1(1971). Panels show double-referenced sensorgrams from a series of five analyte concentrations (12.5, 25, 50, 100, and 200 nM) in twofold dilutions, injected for 120 s with dissociation monitored for 180 s. Each *double red line* represents two overlaid sensorgrams of same concentrations. C4b binding data cannot be fitted to a 1:1 model because of its biphasic binding nature. HuCR1, human complement receptor 1.

As HuCR1 is a receptor for C3b and C4b (31), we examined the affinity of CSL040 for these ligands by surface plasmon resonance (SPR). Both the C3b and C4b used were commercially sourced plasma-derived proteins that under nonreducing conditions appeared predominantly monomeric (Fig. S3). However, a noticeable dimeric fraction was also observed for both C3b and C4b. As shown in Figure 2*A* and Table 2, the affinity of CSL040 to human C3b was determined by SPR to be 264.6 ± 14.4 nM, whereas the control HuCR1(1971) affinity was measured at 385.7 ± 17.1 nM (Fig. 2*B* and Table 2). This difference was small but statistically significant (*p* = 0.0007). Binding of human C4b to CSL040 (Fig. 2*C*) and HuCR1(1971) (Fig. 2*D*) was also observed, but no affinity measurements could be determined as the interaction was biphasic and could not be fitted to a 1:1 model (Table 2). However, there were no qualitative difference in the binding of CSL040 to human C4b compared with HuCR1(1971). These data demonstrate that removal of the LHR-D domain has no or minimal effect on the binding of CSL040 to C3b or C4b and suggests that the improved potency of CSL040 is likely not because of increased ligand-binding affinity.

**Table 2:** Kinetic rate constants and affinity of plasma-derived human C3b and C4b binding to CSL040 or HuCR1(1971)

Receptor	Ligand	*k*_*a*_ (1/Ms)	*k*_d_ (1/s)	*K*_D_ (nM)
CSL040	C3b	2.48 × 10^4^	6.54 × 10^−3^	264.6 ± 14.3
HuCR1(1971)		1.96 × 10^4^	7.54 × 10^−3^	385.7 ± 17.1
CSL040/HuCR1(1971)	C4b	Biphasic (nonfitted)		

*K*_D_ indicated as mean ± SD (*N* = 3); *p* = 0.0007; unpaired *t* test. Values were calculated from sensorgram data fit to a 1:1 binding model for C3b.

HuCR1, human complement receptor 1; *k*_a_, association rate constant; *k*_d_, dissociation rate constant; *K*_D_, equilibrium dissociation constant.

**Figure 3:**
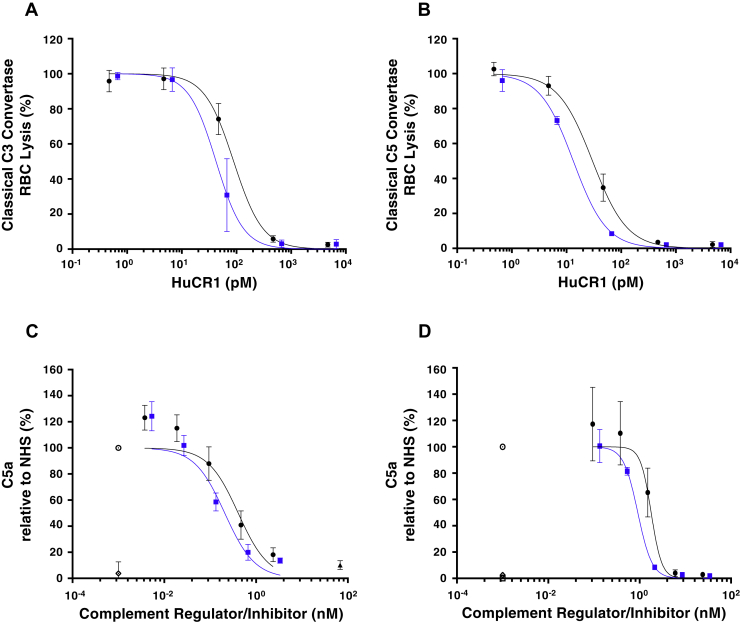
***In vitro* characterization of CSL040 in complement decay acceleration and C5a quantification assays.** CSL040 (*blue squares*) was analyzed in the following *in vitro* assays: the classical pathway C3 (*A*) or C5 (*B*) decay acceleration assay, and in C5a quantification assays following CH50 (*C*) or ApH50 (*D*) hemolytic assays. HuCR1(1971) was used as a comparator (*black circles*). For (*C*) and (*D*), results were calculated relative to NHS (*open circles*), and an anti-C5 mAb (*filled triangle*, panel *C*) was used to show reduced C5a formation. Samples without NHS (*open squares*) and NHS samples containing 20 mM EDTA (*open diamonds*) served as negative controls. *N* = 3 for both CSL040 and HuCR1(1971). Statistically significant differences in values between groups were calculated using an unpaired *t* test. HUCR1, human complement receptor 1; NHS, normal human serum; RBC, red blood cell.

We next sought to examine the complement inhibitory properties of CSL040 in greater detail, using decay acceleration assays to compare the ability of CSL040 and HuCR1(1971) to modify CP-specific C3 or C5 convertases. As shown in Figure 3*A*, CSL040 retained its increased potency compared with HuCR1(1971) in the CP C3 convertase decay acceleration assay *in vitro*, with IC_50_ values determined to be 47.09 ± 15.14 and 90.63 ± 12.93 pM (*p* < 0.05) for CSL040 and HuCR1(1971), respectively. When the C5 convertase decay acceleration assay was employed, an increased potency was again observed for CSL040 compared with HuCR1(1971), with IC_50_ values of 13.32 ± 0.52 and 30.74 ± 4.23 pM (*p* < 0.05), respectively (Fig. 3*B*). Next, C5a anaphylotoxin levels were measured by ELISA in supernatants following CP and AP hemolytic assays, using CSL040 or HuCR1(1971) to inhibit C5a formation. As shown in Figure 3*C*, CSL040 inhibited C5a formation as a byproduct of CP-specific hemolytic activity approximately twofold more efficiently than HuCR1(1971), with IC_50_ values determined to be 0.21 ± 0.03 and 0.44 ± 0.11 nM (*p* < 0.05) for CSL040 and HuCR1(1971), respectively. The results were similar in the AP-specific C5a assay (Fig. 3*D*), with an IC_50_ of 0.91 ± 0.02 nM measured for CSL040 compared with 1.90 ± 0.36 nM (*p* < 0.05) for HuCR1(1971). The ability of CSL040 to cleave C3b in the presence of factor I was also examined. CSL040 was able to act as a cofactor in factor I-mediated C3b cleavage, but there was no difference in the rate of cleavage compared with the HuCR1(1971) control (Fig. S4).

### Biophysical analysis of CSL040

**Figure 4:**
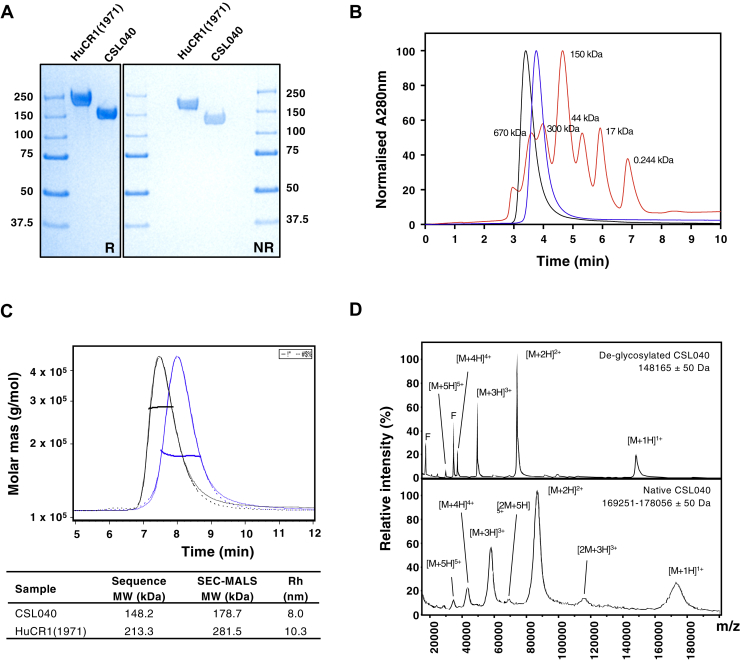
**Biophysical characterization of CSL040.***A*, SDS–PAGE of HuCR1(1971) and CSL040. About 3 μg of each purified protein was electrophoresed under either reducing (R) or nonreducing (NR) conditions alongside molecular weight (MW) standards. Numbers on both sides of the gel refer to the MW sizes of the standards in kilodaltons. *B*, analytical SEC of HuCR1 variants. The purity of CSL040 (*blue*) and HuCR1(1971) (*black*) was analyzed and compared with MW standards (*red*; sizes indicated in kilodaltons above each peak) with all absorbance at 280 nm being normalized. *C*, SEC-MALS analyses of CSL040 (*blue*) and HuCR1(1971) (*black*). The chromatogram shows the normalized refractive index (RI) signal (*solid lines*) overlaid with the light scattering (LS) signal (*dotted lines*). The *horizontal lines* display the weight-averaged molar mass of the eluted peak. The table below shows the predicted and SEC-MALS–derived MW of each protein in kilodaltons, as well as the hydrodynamic radius (Rh) in nanometers (nm). *D*, comparison of MALDI-TOF mass spectrometry spectra for PNGase F-treated deglycosylated CSL040 (*top panel*) and native CSL040 (*bottom panel*). F denotes the position of the signal corresponding to PNGase F single and doubly charged forms. [M + nH]^n+^ denotes the molecular mass ion, whereby M is the mass peak observed, H is the proton adduct, and + being the number of charges carried by the ion. Estimated glycosylated mass ranges for CSL040 were based on the full width at half maximum of the triple-charged form. HUCR1, human complement receptor 1.

**Figure 5:**
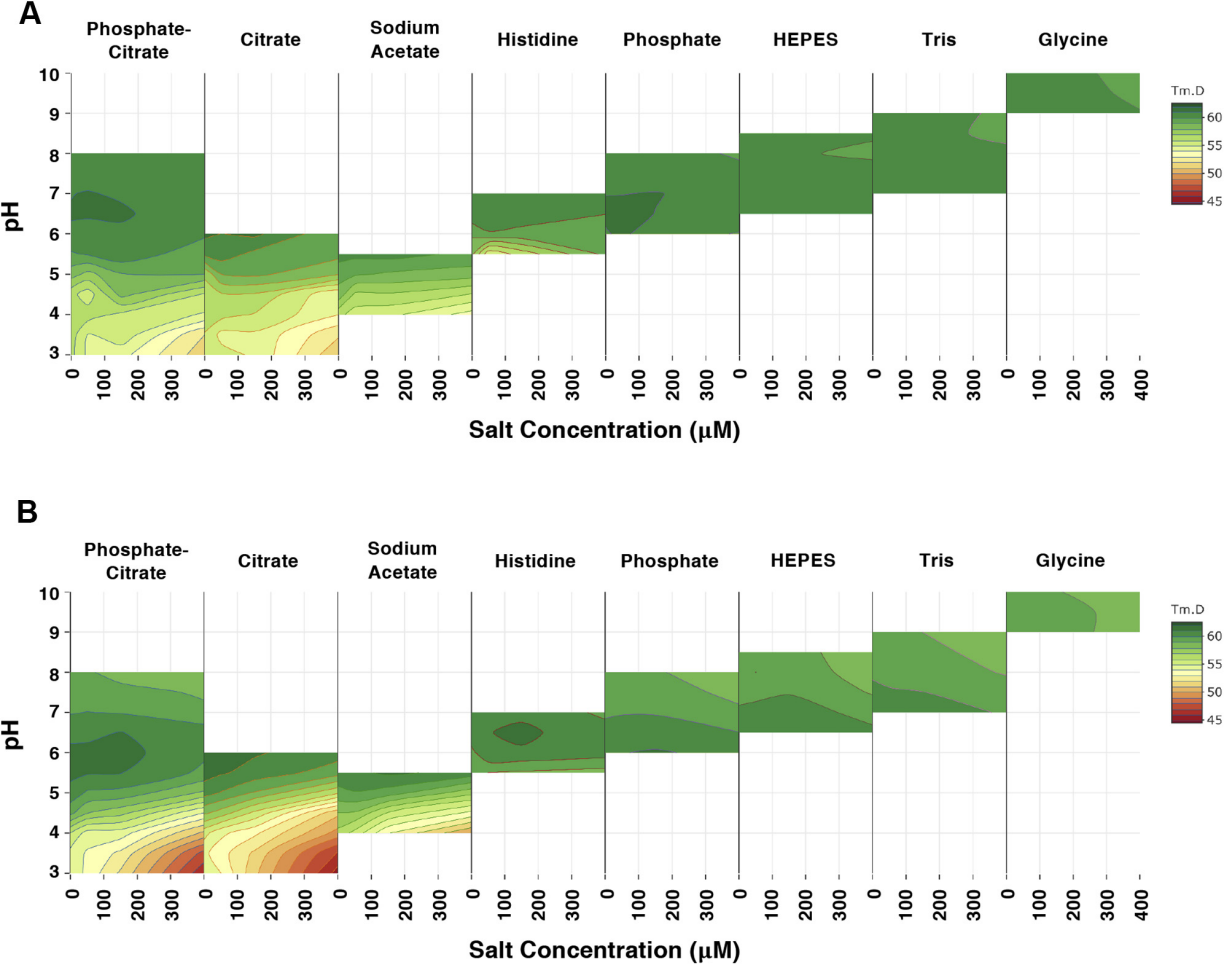
**Comparison of HuCR1 protein thermal stability by differential scanning fluorimetry**. *A*, CSL040. *B*, HuCR1(1971). Each column represents a different buffer condition. Within each *box*, a range of pH values (3.0–10.0) are shown on the *y*-axis and a range of NaCl concentrations (0–500 mM) on the *x*-axis. For each condition (HuCR1 in a particular buffer, pH, and NaCl concentration), the melting temperature is depicted as a color based on the key, with the contour lines representing a 1 °C change.

We next analyzed the biophysical characteristics of CSL040. Although both unpurified (Fig. S1) and purified (Fig. 4*A*) CSL040 and HuCR1(1971) were shown to be monomeric by SDS–PAGE, size-exclusion chromatography (SEC) analysis showed that both CSL040 and HuCR1(1971) migrated as single peaks with apparent molecular weight (MW) in excess of 300 kDa (Fig. 4*B*), suggesting that they may exist either as oligomers or that they both possess a large hydrodynamic radius. To resolve this discrepancy, SEC with multiangle light scattering (SEC-MALS) was performed on both purified CSL040 and HuCR1(1971). As shown in Figure 4*C*, the results of the SEC-MALS analysis confirmed that both CSL040 and HuCR1(1971) are monomers with minimal levels of aggregation and with significant glycan content. The measured MW of CSL040 was determined by SEC-MALS to be 178.7 kDa, 30.5 kDa larger than the predicted MW of 148.2 kDa (Fig. 4*C*). The size discrepancy (17.1% of the total MW) was determined through peptide:N-glycosidase (PNGase) treatment of CSL040, followed by MALDI-TOF mass spectrometry to be primarily because of N-linked glycosylation (Fig. 4*D*). In contrast, the parental control HuCR1(1971) was a much larger, 281.5 kDa, protein as measured by SEC-MALS (Fig. 4*C*), reflecting a total glycan content of 24.2%. Interestingly, the hydrodynamic radius of CSL040 was also 20% smaller than HuCR1(1971) (Fig. 4*C*), although both were large (10.3 and 8.0 nm, respectively), indicating that they are likely elongated in shape and explaining the large apparent MW observed by SEC (Fig. 4*B*). The stability of CSL040 was then analyzed using differential scanning fluorimetry, an assay for measuring thermal stability. The results of this assay are shown in Figure 5, and CSL040 (Fig. 5*A*) appears to be not only stable across a wide range of pH values and NaCl concentrations in the eight buffer systems tested but generally more stable than the HuCR1(1971) control across the same conditions (Fig. 5*B*).

### Sialylation is an important mechanism of CSL040 clearance *in vivo*

**Figure 6:**
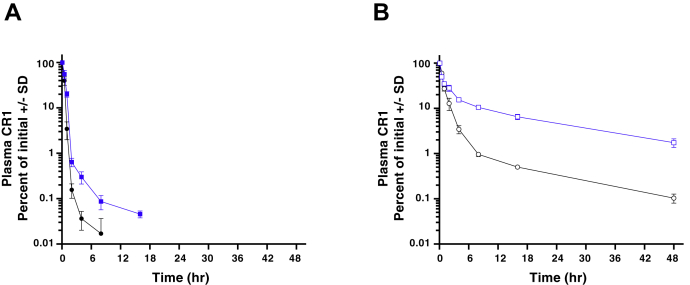
**Pharmacokinetics of CSL040 *in vivo*.** Wildtype mice were administered 30 mg/kg CSL040 (*blue squares*) or a molar equivalent 43 mg/kg HuCR1(1971) (*black circles*) as an i.v. bolus and CR1 concentrations from three serum samples per set time point were measured by a CR1-specific ELISA and plotted as a percentage of the first dose. The recombinant protein used was generated with either high asialo content (*filled squares*, *circles*; panel *A*) or low asialo content (*open squares*, *circles*; panel *B*). See Table 3 for N-glycan content of each test article. CR1, complement receptor 1.

The pharmacokinetic properties of CSL040 *in vivo* were then assessed in wildtype mice (a crossreactive species), comparing its effects to HuCR1(1971) using equivalent molar doses of 30 and 43.2 mg/kg, respectively. As CSL040 is a heavily glycosylated protein, we surmised that sialylation may play a role in its *in vivo* clearance and so generated versions with both low and high asialo content (Table 3). The control HuCR1(1971) proteins used had similar glycoprofiles with those of CSL040 (Table 3). Comparison of either protein with low and high asialo content shows that when CR1 asialo content is reduced *via* coexpression of ST3GAL3 and B4GALT1, that proportion of N-glycans appears mainly shifted to the disialo type and to a lesser extent the trisialo type (Table 3). The *in vitro* potency of CSL040 was unaffected by its asialo content (Fig. S5). CSL040 with a high asialo content of 74.5% was cleared rapidly following administration in mice (Fig. 6*A*); this clearance was slower compared with the control HuCR1(1971) protein with 77.6% asialo content. In contrast, CSL040 with a low asialo (24.1%) content showed a dramatically reduced rate of clearance *in vivo* (Fig. 6*B*), with superior pharmacokinetic properties compared with the control HuCR1(1971) with 28.0% asialo content, also observed. Analysis of the data was performed using a noncompartmental analysis, the results of which are shown in Table 4. These data confirmed that low asialo CSL040 has superior pharmacokinetic properties compared with high asialo CSL040 (clearance 17.4 ± 0.8 and 126.5 ± 15.8 ml/kg/h; mean residence time [MRT] 16.4 ± 1.8 and 0.77 ± 0.08 h; terminal half-life 15.7 ± 1.5 and 9.0 ± 1.5 h, respectively; Table 4) and also to HuCR1(1971) *in vivo*, regardless of its asialo content (Table 4). The differences between CSL040 and HuCR1(1971) were statistically significant for both MRT and terminal half-life, regardless of asialo content (Table 4). Experiments directly comparing CSL040 with high and low asialo content as mentioned previously showed similar results (Fig. S6).

**Table 3:** Proportion of released N-glycans from different batches of Expi293F-derived CSL040 or HuCR1(1971), showing asialo (neutral), monosialo, disialo, trisialo, and four or more (tetrasialo+) sialic acids

N-glycan type	CSL040 (%)	HuCR1(1971) (%)	CSL040 (%)	HuCR1(1971) (%)
Asialo	24.1	28.0	74.5	77.6
Monosialo	22.8	25.9	21.1	17.6
Disialo	41.9	37.3	3.7	4.3
Trisialo	9.1	6.3	0.6	0.4
Tetrasialo+	2.1	2.5	0.1	0.1

Each number is expressed as a percentage of the total glycan content measured. The batches of CSL040 and HuCR1(1971) with the lower asialo content were generated by coexpression of B4GALT1 and ST3GAL3.

HuCR1, human complement receptor 1.

**Table 4:** Noncompartmental analysis of CR1 concentration measurements over time, following administration of 30 mg/kg CSL040 or 43.2 mg/kg HuCR1(1971) with varying asialo levels administered to *N* = 3 mice in *in vivo* pharmacokinetic assays

Protein	N-glycan asialo (%)	AUC_infinity (h^a^ μg/ml)	AUC_last (h^a^μg/ml)	% extrapolated	Clearance (ml/kg/h)	MRT (h)	Terminal half-life (h)	Vc (ml/kg)	Vss (ml/kg)	Vz (ml/kg)	Cmax (μg/ml)	IVR (%)
HuCR1(1971)	28.0	665 ± 30.5^b^	655 ± 28.5^b^	1.5 ± 0.3^c^	65.1 ± 3.0^b^	4.2 ± 0.3^b^	12.8 ± 0.9^a^	75.9 ± 4.0	271 ± 8.1	1200 ± 30.0^c^	571 ± 29.5^a^	52.8 ± 2.7
CSL040	24.1	1730 ± 85.0	1570 ± 57.7	9.1 ± 1.9	17.4 ± 0.8	16.4 ± 1.8	15.7 ± 1.5	67.4 ± 5.5	284 ± 22.0	395 ± 28.4	447 ± 36.0	59.6 ± 4.8
HuCR1(1971)	77.6	195 ± 9.3	195 ± 9.2	0.09 ± 0.05^a^	153.8 ± 7.3	0.30 ± 0.05^c^	2.5 ± 1.4^a^	69.7 ± 3.8	46.2 ± 5.5	540 ± 306^a^	431 ± 23.3	57.5 ± 3.1
CSL040	74.5	240 ± 30.5	238 ± 30.7	0.65 ± 0.25	126.5 ± 15.8	0.77 ± 0.08	9.0 ± 1.5	84.7 ± 11.0	98.2 ± 19.0	1653 ± 376	358 ± 44.2	47.8 ± 5.9

Values are expressed as mean ± SD. Definitions of each parameter are found in the Experimental procedures section.

AUC, area under the curve; Cmax, peak concentration of test article after administration; CR1, complement receptor 1; HuCR1, human complement receptor 1; IVR, *in vivo* recovery; MRT, mean residence time; Vc, central volume of distribution; Vss, steady-state volume of distribution; Vz, terminal phase volume of distribution.

a *p* < 0.05.

b *p* < 0.0005 (unpaired *t* test) for low asialo CSL040 compared with low asialo HuCR1(1971), and high asialo CSL040 compared with high asialo HuCR1(1971).

c *p* < 0.005.

### CSL040 attenuates kidney damage in a murine model of GN

**Figure 7:**
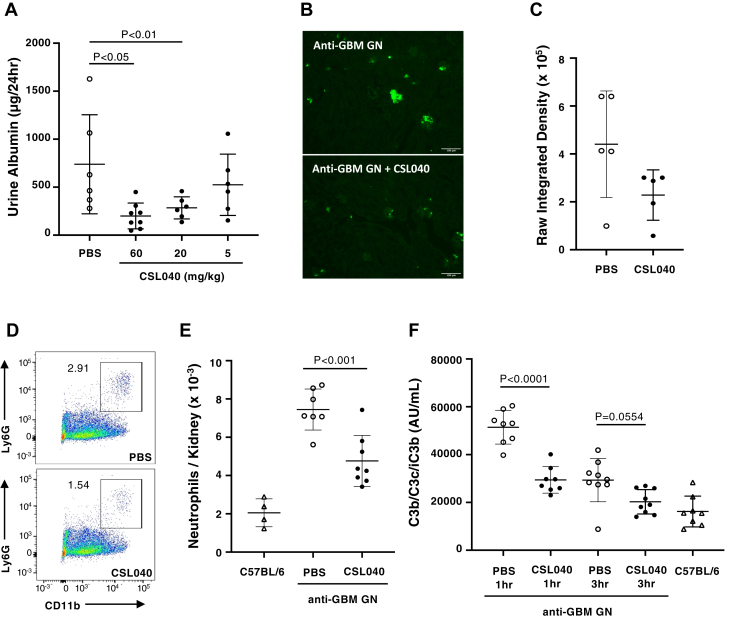
**The effect of CSL040 in an attenuated-passive anti-GBM glomerulonephritis mouse model.** Glomerulonephritis was induced in C57BL/6 mice by i.v. injection of rabbit anti-GBM polyclonal antibody followed 6 days later by i.p. administration of MsαRb antibody. CSL040 (*filled circles*) or a PBS control (*open circles*) was given by i.p. administration 1 h prior to MsαRb. Naive C57BL/6 mice (*open triangles*) were used as an additional control. Urine was collected using metabolic cages, and mice were sacrificed after 24 h (for albuminuria and complement deposition) or 1 to 24 h (neutrophils staining in kidneys and measurement of plasma complement components). *N* = 6 to 8 per group. *A*, urine albumin levels in mice treated with PBS or CSL040 at 5, 20, or 60 mg/kg. *B*, intraglomerular infiltration of neutrophils 3 h after MsαRb administration was diminished by 60 mg/kg CSL040 as shown by the representative images of immunofluorescence staining of mouse kidneys (magnification 100×; the bar represents 100 μm). *C*, quantification of raw integrated density. *D*, infiltrating neutrophils in kidney were quantified by flow cytometry using the Ly6G and CD11b markers. The number next to the gate (*outlined with a box*) is neutrophils as a percentage of CD45^+^ cells in the plot of one representative mouse. *E*, neutrophil numbers from mice in each group were calculated from flow cytometry. *F*, effect of PBS or CSL040 (60 mg/kg) treatment on the plasma levels of complement activation fragment C3b/C3c/iC3b after induction of glomerulonephritis. Data shown are mean ± SD. Statistically significant differences in values between groups were calculated using ordinary one-way ANOVA with the Tukey's test for multiple comparisons. GBM, glomerular basement membrane; GN, glomerulonephritis.

Having shown sialylation is important to CSL040 clearance, we used CSL040 with low asialo content to test its potency *in vivo* in the attenuated passive anti-glomerular basement membrane (GBM) GN mouse model, in which complement plays a key role in disease development. Mice were injected intravenously with a subnephritogenic dose (1 mg) of rabbit antimouse GBM. Six days later, the animals were injected intraperitoneally with mouse anti-rabbit monoclonal immunoglobulin G (IgG) (MsαRb). Consequently, animals injected with anti-GBM antibody and MsαRb also showed a marked albuminuria development (Fig. 7*A*). In contrast, mice that received PBS or anti-GBM antibody alone did not develop albuminuria (Fig. S7). One hour prior to the injection of the secondary antibody, mice were intraperitoneally injected with either PBS control or CSL040 at 5, 20, or 60 mg/kg. Mice treated with 60 or 20 mg/kg CSL040 developed significantly lower albuminuria compared with the PBS controls (Fig. 7*A*), suggesting that CSL040 can effectively attenuate kidney damage *in vivo*. CSL040 administered at 5 mg/kg was ineffective (Fig. 7*A*). Comparison of neutrophil infiltration (Fig. 7, *B*–*C*) between vehicle control mice and CSL040-treated mice showed an attenuated inflammatory response in the kidneys of the CSL040-treated mice. The reduction of renal neutrophil infiltration following CSL040 treatment was further confirmed by flow cytometry analysis (Fig. 7, *D*–*E*). Anti-GBM GN did not result in significant consumption of systemic complement as measured by C3 levels (Fig. S8). Disease development in the anti-GBM GN model resulted in significant increase in plasma C3b/C3c/iC3b levels at 1 and 3 h after MsαRb administration (Fig. 7*F*), declining and returning to baseline at 24 h as compared with the nondisease control mice (Fig. S9). The timing of complement activation in the model correlates with that of neutrophil infiltration into the kidneys. CSL040 significantly decreased plasma C3b/C3c/iC3b levels compared with the vehicle control at 1 h following MsαRb administration (Fig. 7*F*). At 3 h, plasma C3b/C3c/iC3b levels were also reduced by CSL040 (Fig. 7*F*), but this reduction was not found to be statistically significant (*p* = 0.0554). Together, these data show that CSL040 is able to dose dependently inhibit disease development in an animal model of complement-mediated renal damage.

## Discussion

Soluble HuCR1 is a highly potent complement inhibitor with evidence of clinical efficacy (57, 59). Previous studies have examined the relative contributions of the LHR domains of HuCR1 to the inhibition of complement activity, either as single-expressed LHR domains or as a limited set of LHR combinations (44, 60). However, these studies were limited to comparing hemolytic activity at a single (1 μM) dose. The present work expands on this by testing every sequential LHR domain combination (Fig. 1) in dose–response inhibition studies for all complement pathways, both hemolytic and Wieslab (Table 1). In doing so, we have identified a novel therapeutic candidate (CSL040), a soluble HuCR1 fragment containing the LHR-A, LHR-B, and LHR-C domains of HuCR1. CSL040 was shown to be the most potent inhibitor of all complement pathways compared with all other soluble HuCR1 fragments tested, as well as full-length soluble HuCR1(1971). Our studies comparing the truncated HuCR1 fragments also show that the bulk of the inhibitory activity of soluble HuCR1 is located toward the N-terminal end of the molecule, and that multiple LHR domains are needed to confer the most potent inhibition of complement activation *in vitro*. We confirm previous data showing that the LHR-D domain has no inhibitory activity (44, 60), despite reports that the LHR-D domain interacts with complement components C1q and mannose-binding lectin (61, 62).

The mechanism explaining the increased potency of CSL040 is unclear. Multiple studies have demonstrated that the C3b-binding sites on HuCR1 are located in LHR-B and LHR-C (31, 63, 64, 65, 66, 67, 68), and that the C4b-binding sites are located in LHR-A, LHR-B, and LHR-C (64, 65, 69, 70). This has clear implications for factor I cofactor activity of HuCR1, which parallels C3b/C4b ligand binding (65, 66, 68), and also for the decay acceleration activity of HuCR1, which is likewise located in LHR-A, LHR-B, and LHR-C (31, 71). Indeed, in our hands, the absence of LHR-D in CSL040 only conferred a small increase in affinity to C3b (Table 2). It is not clear whether this increase in affinity of CSL040 to C3b compared with HuCR1(1971) has any direct link to its activity. However, CSL040 is more potent compared with HuCR1(1971) in decay acceleration activity assays (Fig. 3, *A*–*B*), in which potency is linked to ligand binding (31). The affinity of CSL040 to C3b measured in our study is derived from data that are well described by a simple 1:1 kinetic model, providing confidence in the measurement, and differing from previously reported measurements of 140 and 69 nM, which did not fit a 1:1 model (72, 73). We did attempt the reverse SPR experiment, immobilizing C3b/C4b on the biosensor surface, but we found the immobilized ligand to be only 20% active (compared with more than 70% for immobilized CR1), most likely because of chemical modification during amine coupling. This is a similar phenomenon reported by others, with observed surface activities of 5 to 20% (74), and is generally undesirable for SPR experiments. In this format, we measured apparent affinities for CR1 to immobilized C3b and C4b at approximately 1.5 and 4.9 μM, respectively (data not shown), somewhat weaker than previously published (74). However, this discrepancy could be due to the lower surface density of immobilized ligand used to reduce avidity (230RU) or to differences in the top concentration of CR1 employed.

**Figure 8:**
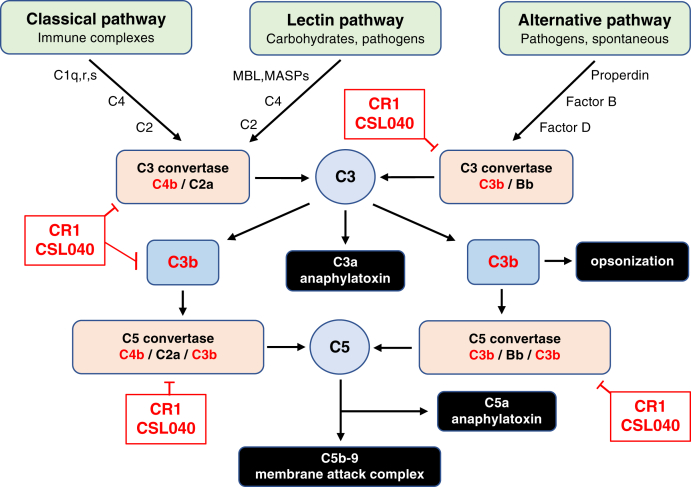
**Involvement of CR1/CSL040 in complement pathway inhibition.** The three pathways of the complement systems are the classical, lectin, and alternative pathways, each activated by specific mechanisms. All pathways converge at the level of C3 before diverging to form the key complement end products: the C3a and C5a anaphylatoxins that mediate inflammatory processes; the opsonin C3b; and the lytic membrane attack complex. The activated fragments of C3 and C4, C3b and C4b, are key components in all three complement pathways, and CR1/CSL040 (*red boxes*) can bind both C3b and C4b, thereby inhibiting further complement activation. One mechanism is *via* CR1/CSL040 displacement of C2a or Bb in the C3 and C5 convertases (decay acceleration); the other is promotion of factor I-mediated cleavage of C3b/C4b into inactive fragments (cofactor activity). CR1, complement receptor 1.

CSL040 retained the ability to cleave C3b, but the assay used was not sufficiently sensitive to determine anything other than qualitative similarities compared with HuCR1(1971). In order to eliminate the possibility that the HuCR1(1971) used in these experiments was impure or was structurally unsound compared with CSL040, the biophysical properties of CSL040 and HuCR1(1971) were compared. Both proteins were found to be pure and monomeric (Fig. 4), discounting this possibility as an explanation of the differences in *in vitro* activity. Alterations in the folding of CSL040 induced by the removal of LHR-D may also play a role in its improved activity, perhaps by improving accessibility to ligand-binding sites, or by increasing its stability, an effect noted in the differential scanning fluorimetry experiments (Fig. 5). Interestingly, constrained scattering modeling studies of the solution structure of soluble CR1 have suggested folded back arrangements of SCR domains, with pronounced bends at the N-terminal ends of LHR-C and LHR-D (75). The absence of LHR-D in CSL040 may reduce this folding back phenomenon, thereby impacting and explaining its potency. Indeed, a related complement protein with similar arrangement of 20 SCR domains, factor H, has been shown to fold back upon itself to engage C3b ligand, which then results in further unfolding and exposure of additional ligand-binding sites (76, 77). The *in vitro* and biochemical studies presented in this article thus describe a mechanism by which CSL040 is able to exert its inhibitory effects on the complement system; this is shown in Figure 8. In an almost identical fashion to CR1 (whether soluble or transmembrane), CSL040 is able to bind its ligands C3b and/or C4b and inhibit activation of all three complement pathways, either by its decay acceleration activity whereby the C3/C5 convertases are destabilized or acting as a cofactor for factor I-mediated cleavage of C3b/C4b (Fig. 8).

The pharmacokinetic profile of a glycoprotein can often determine its success or failure as a potential therapeutic, and sialylation is one of the most important factors that can modulate glycoprotein half-life *in vivo*, the lack of which is clearly deleterious (78, 79, 80). TP-10, the full extracellular domain of HuCR1 previously developed as a soluble therapeutic, has been shown from studies in multiple species including humans (43, 49, 57) to have a relatively short half-life compared with antibody-based therapies (81), limiting its use to acute indications. We examined the role of sialylation in modulating the half-life of CSL040, with the aim of improving its pharmacokinetic parameters and therefore its potential as a therapeutic. CSL040 protein with a high asialo content (74.5%) generated in Expi293F cells displayed a similar half-life in mice (Fig. 6*A*) to that of HuCR1(1971), which is similar to the previously described sCR1/TP-10 molecule (43). However, a CSL040 with much lower (24.1%) asialo content was generated by coexpression of B4GALT1 and ST3GAL3 in Expi293F cells, and this low asialo CSL040 displayed a dramatically improved pharmacokinetics in mice (Fig. 6*B*). The importance of the sialyltransferase ST3GAL3 in mediating alpha 2,3-linked sialylation has previously been demonstrated (82), and other studies have shown that coexpressing ST3GAL3 with the precursor galactosyltransferase B4GALT1 both increases sialylation and attenuates clearance of recombinant glycoproteins (83, 84, 85). It is likely that the mechanism by which the high asialo CSL040 is cleared is *via* recognition and engagement of the asialoglycoprotein receptor to exposed terminal galactose residues on glycans, which then leads to endocytosis and elimination from the circulation (86, 87, 88, 89). Taken together, these data also suggest that the therapeutic potential of CSL040 can be further optimized by manipulation of its asialo levels.

The attenuated passive anti-GBM mouse model employed in these studies has been shown through the use of selected knockout mice to have both significant complement and Fc-gamma receptor involvement (90). In terms of complement involvement, this model appears to mirror the characteristics of patients with anti-GBM disease, with a study showing complement deposits in all the diseased kidneys tested (91). We demonstrated a dose-dependent attenuation of GN-associated albuminuria in the mouse anti-GBM model using CSL040 compared with control (Fig. 7*A*), which may be attributed to the inhibition of complement activation in some or all pathways by CSL040. Upon complement activation, the generated C5b molecule is involved in the formation of C5b-9, the end product of the terminal complement pathway, whereas C5a and C3a molecules are chemoattractants for neutrophils. CSL040 treatment inhibited glomerular neutrophil infiltration (Fig. 7, *B*–*E*). The role of C5b-9 in this murine GN model is unclear, and additional studies will be required to reveal whether it is involved in the mechanism of kidney damage. The C3b/C3c/iC3b ELISA, a sensitive tool for the assessment of systemic complement activation, was applied to assess and characterize the experimental mouse passive GN observed in this model. C3b/C3c/iC3b fragments in mouse plasma increased rapidly after the administration of MsαRb for disease induction, peaked at 1 h, and returned to baseline by 24 h after MsαRb administration. Complement activation dynamics correlate with renal neutrophil infiltration, which peaked at 3 h post-MsαRb administration, suggesting a role for local complement activation promoting kidney inflammation with neutrophil infiltration. CSL040 was revealed to significantly attenuate C3b/C3c/iC3b levels at 1 h after disease induction.

In conclusion, we have identified and characterized CSL040, a novel soluble truncated version of HuCR1. Through optimization of its asialo content to maximize *in vivo* half-life, CSL040 is likely to have superior therapeutic potential to TP-10 in a number of clinical indications where complement-mediated damage is a significant contributor to disease pathophysiology.

## Experimental procedures

### Generation of complementary DNA expression plasmids

HuCR1 complementary DNA (GenBank accession no.: NP_000564), human B4GALT1 (GenBank accession no.: NP_001497), and ST3GAL3 (GenBank accession no.: NP_006270) were codon optimized for human expression and synthesized by GeneArt (Thermo Fisher Scientific, USA). Full-length and truncated soluble HuCR1 variants with in-frame C-terminal 8× His-tags and double stop codons were generated using standard PCR-based mutagenesis techniques. A version of HuCR1(1392)/CSL040 without a C-terminal His-tag was also generated. For N-terminal HuCR1 truncation mutants, an exogenous signal peptide, comprising amino acids 1 to 19, of ceruloplasmin (GenBank accession no.: NP_000087) was employed. Each complementary DNA was generated with a Kozak consensus sequence (GCCACC) immediately upstream of the start codon and ligated into pcDNA3.1 (Thermo Fisher Scientific). Large-scale preparations of plasmid DNA were carried out using QIAGEN plasmid giga kits according to the manufacturer's instructions (QIAGEN, Germany). The nucleotide sequences of all plasmid constructs were verified by sequencing both strands using BigDye Terminator Version 3.1 Ready Reaction Cycle Sequencing (Thermo Fisher Scientific, USA) and an Applied Biosystems 3130xl Genetic Analyzer (Applied Biosystems, USA).

### Cell culture and transient transfections

Expi293F cells and the mammalian expression vector pcDNA3.1 were obtained from Thermo Fisher Scientific, USA. Cells were cultured in Expi293 expression medium (Thermo Fisher Scientific, USA). All tissue culture media were supplemented with antibiotic–antimycotic solution (Thermo Fisher Scientific, USA), and cells were maintained at 37 °C in incubators with an atmosphere of 8% CO_2_. Transient transfections of expression plasmids using Expi293F cells were performed using Expi293 expression system according to the manufacturer's recommendations (Thermo Fisher Scientific, USA). All proteins were generated as highly sialylated entities *via* cotransfection of HuCR1-encoding plasmids with plasmids encoding B4GALT1 and ST3GAL3 at a DNA ratio of 94:3:3, respectively. Poorly sialylated recombinant protein was generated as previously, but without cotransfection of B4GALT1/ST3GAL3.

### Purification of HuCR1

For the purification of 8× His-tagged HuCR1 proteins, culture supernatant from transiently transfected Expi293F cells was loaded directly onto nickel sepharose HisTrap excel affinity resin (GE Healthcare, USA) pre-equilibrated with 20 mM NaH_2_PO_4_, 500 mM NaCl, 10 mM imidazole, and pH 7.4. The resin was washed with 20 mM NaH_2_PO_4_, 500 mM NaCl, 25 mM imidazole, pH 7.4, then HuCR1 was block eluted with 20 mM NaH_2_PO_4_, 500 mM NaCl, 500 mM imidazole, and pH 7.4, with the collection of eluted protein based on absorbance at 280 nm. Protein collected was loaded onto a HiLoad 26/60 superdex200 prep grade column (GE Healthcare, USA) pre-equilibrated in PBS (137 mM NaCl, 27 mM KCl, 8.1 mM Na_2_HPO_4_, 1.15 mM KH_2_PO_4_, and pH 7.4) to separate contaminating proteins and buffer exchange HuCR1 into PBS. Purified HuCR1 was concentrated using Amicon ultra centrifugal filters (MilliporeSigma, USA) with a MW cutoff of 50 kDa, sterile filtered, and stored at −80 °C. Untagged CSL040 was purified by cation exchange chromatography followed by SEC in 1× PBS and pH 7.4. CSL040 culture supernatant was diluted one in three with 50 mM MES with pH 5.5, adjusted to pH 6.0 with 1 M HCl and 0.22 μm filtered prior to loading onto a HiPrep SP FF column (GE Healthcare, USA) pre-equilibrated with 50 mM MES with pH 6.0. The resin was then washed with six column volumes of the same. CSL040 was eluted with 50 mM MES, 500 mM NaCl, and pH 6.0. Postelution steps were then carried out as per His-tagged protein.

### Wieslab complement inhibition assays

Wieslab complement assays (Euro Diagnostica AB, Sweden) were performed according to the manufacturer's recommendations. Briefly, each HuCR1 protein was serially diluted in PBS buffer to ∼5× the final concentration in a 96-well plate. About 50 μl of each protein or PBS alone was then added to prediluted human serum in the appropriate assay diluent and incubated for 30 min incubation at RT. Samples were transferred to the assay plate in duplicate, incubated for 1 h at 37 °C (with no CO_2_), then wells emptied, and washed 3 times. C5b-9 was detected using alkaline phosphatase–conjugated antibody, which was incubated for 30 min at RT. Bound antibodies were detected using alkaline phosphatase substrate solution and incubated for 30 min at RT. Absorbance at 405 nm was read using the EnVision plate reader (Perkin Elmer, USA). Raw values were expressed as a percentage of C5b-9 formation by the serum and PBS only control (*i.e.*, 100% C5b-9 formation). Results were analyzed in GraphPad Prism for IC_50_ values using a log(inhibitor) *versus* response; variable slope (four parameters) fit. Unpaired *t* tests were also performed in GraphPad Prism to compare the IC_50_ values of each HuCR1 fragment to the parental HuCR1(1971) IC_50_.

### Hemolytic assays

For the complement CP-specific assay (CH50), sheep erythrocytes (Acila Schweiz AG, Switzerland) were sensitized with rabbit antisheep antibodies (hemolysin; bioMérieux, France) and diluted to 4 × 10^8^ cells/ml gelatin veronal buffer (GVB)^++^ (GVB, 0.15 mM CaCl_2_, 0.5 mM MgCl_2_; CompTech, USA). To assess the inhibition of the CP, HuCR1 variants were preincubated in 1 of 40 diluted normal human serum (NHS; Swiss Blood Donation Center, Switzerland) for 30 min at RT, added to the erythrocytes at a 1/1 (v/v) ratio, and incubated during 1 h at 37 °C in a microtiter plate—shaking device. After adding ice-cold GVB with 10 mM EDTA (Rockland Immunochemicals, USA) and centrifugation (5 min at 1250*g*, 4 °C), hemolysis was determined in the supernatant by measuring the absorbance of released hemoglobin at 412 nm. For the complement AP-specific (ApH50) assay, rabbit erythrocytes (Jackson Laboratories, Boston, USA) were washed and diluted to 2 × 10^8^ cells/ml GVB/MgEGTA (GVB, 5 mM MgEGTA; CompTech, USA). To assess the inhibition of the AP, prediluted HuCR1 variants were preincubated in 1/6 diluted NHS for 30 min at RT, added to the erythrocytes at a 2/1 (v/v) ratio, and incubated during 1 h at 37 °C in a microtiter plate–shaking device. After adding ice-cold GVB with 10 mM EDTA and centrifugation (10 min at 1250*g*), hemolysis was determined in the supernatant by measuring the absorbance of released hemoglobin at 412 nm using the EnVision plate reader (Perkin Elmer, USA). For both assays, cells incubated with NHS and the corresponding buffers served as 100% lysis controls. The inhibition of lysis by HuCR1 was calculated relative to this control. Results were analyzed in GraphPad Prism as aforementioned.

### CP C3/C5 convertase decay acceleration assays

The C3/C5 convertases were assembled on hemolysin-sensitized sheep red blood cells (prepared as described previously) by the addition of C1 (6 μg/7.5 × 10^8^ cells; 15 min at 30 °C), C4 (10 μg/7.5 × 10^8^ cells; 15 min at 30 °C), C2 (1 μg/7.5 × 10^8^ cells; 4 min at RT), and C3 (15 μg/7.5 × 10^8^ cells; 4 min at RT; C5 convertase only). All complement components were obtained from CompTech, USA. Prediluted HuCR1 is mixed with sheep red blood cells carrying the C3 or the C5 convertases and incubated during 10 min at 30 °C. Afterward, premixed C3–C9 (250 ng/1.875 × 10^7^ cells) or C4–C9 (C5 convertase assay; 250 ng/1.25 × 10^8^ cells) was added and incubated during 30 min at 37 °C. After centrifugation, supernatants were collected, and free hemoglobin of lysed cells is measured at 414 nm using the EnVision plate reader (Perkin Elmer, USA). Results were analyzed in GraphPad Prism for IC_50_ values as mentioned previously.

### Complement fragment C5a quantification ELISAs

MicroVue complement fragment kits (Quidel Corporation, USA) were used for the quantification of C5a activation fragments in supernatants originating from CH50 or ApH50 assays, respectively. The assays were performed according to manufacturer's instructions. In brief, supernatants from CH50 assays were applied undiluted to the assay plate and incubated during 60 min at RT. Supernatants containing NHS only served as a positive control for complement activation. NHS + 20 mM EDTA served as a negative control. After washing, the conjugate was added and incubated during 60 min at RT. The plates were washed again, and 100 μl of substrate was added for 15 min at RT. The enzymatic reaction was stopped by the addition of 100 μl of stop solution, and absorbance was measured at 450 nm using the EnVision plate reader. For the calculation of the relative content of C5a in the supernatants, the NHS control (positive control) was set as 100%. The supernatants originating from ApH50 assays were analyzed as described previously.

### Cofactor activity assay

About 3 μg C3b and 60 ng factor I (CompTech, USA) were incubated for 60 min at 37 °C with 0 to 160 ng HuCR1. After incubation, 2× prediluted NuPAGE LDS Sample Reducing Agent (Thermo Fisher Scientific, USA) was added at a 1/1 (v/v) ratio and heated at 60 °C for 15 min. Reduced samples were then run on 8% BisTris Plus SDS gels (Thermo Fisher Scientific, USA) to separate C3b α chain from its fragments. A Coomassie stain was then performed according to manufacturer's instructions, using the GelCode Blue Stain Reagent (Thermo Fisher Scientific, USA). For the quantification of the cofactor activity of the analyzed HuCR1 variants, stained gels were imaged using an ImageQuant LAS 4000 Luminescent Image Analyzer (GE Healthcare, USA), and the intact C3 α chain was quantified using GeneTools densitometry Software (SynGene, UK). For the calculation of uncleaved C3b α chain, a control sample (C3b incubated with factor I only) was run in parallel and set as 100% C3b α chain.

### Biosensor analysis

Binding kinetics were measured by SPR at 37 °C using a Biacore T200 biosensor docked with an nitrilotriacetic acid (NTA) (carboxymethylated dextran matrix preimmobilized with NTA) sensor chip (GE Healthcare, USA). The sensor surface was preconditioned with a 1 min pulse of 350 mM EDTA and charged for 1 min with 0.5 mM NiCl_2_ before equilibration with running buffer (10 mM Hepes, 150 mM NaCl, 0.1% bovine serum albumin, and pH 7.3) at the beginning of each cycle. His-tagged recombinant CSL040 and HuCR1(1971) were captured on the NTA surface to 75 to 100 resonance units. For kinetic experiments, human C3b or C4b (15.6–1000 nM; Complement Technology, USA) samples were prepared in running buffer and injected over the CR1 surface in duplicate and random order for 150 s with dissociation monitored for 180 s. At the end of each cycle, the surface was regenerated with a 60 s injection of 350 mM EDTA followed by a buffer wash. All SPR data were processed and analyzed using BIAevaluation software (GE Healthcare, USA). Responses from the reference surface (in which no CR1 was captured) were subtracted from the active surface to produce reference-subtracted data. Reference-subtracted responses from buffer blank injections were subtracted from the resultant sensorgrams to produce double-referenced data. Data were fitted to a 1:1 interaction model including a term for mass transport to obtain binding parameters. The *R*_max_ value was fitted locally to account for slight differences in the capture levels of CR1 with other binding parameters fitted globally.

### SEC and SEC-MALS

HuCR1 proteins were loaded onto a Superdex200 increase 5/150gl column (GE Healthcare, USA) pre-equilibrated with PBS with pH 7.4 using an Agilent 1260 infinity II HPLC system (Agilent Technologies, USA). Elution was performed using an isocratic gradient at 0.4 ml/min in PBS with pH 7.4 at room temperature monitoring UV absorbance at 280 nm. SEC-MALS was performed using an Agilent 1200 series HPLC in series with a Wyatt DAWN Heleos II EOS MALS detector and Wyatt Optilab T-rEx RI detector (Wyatt Technology Corporation, USA). For analysis, approximately 20 μg of HuCR1 protein was injected onto a Superdex 200 Increase 5 × 150 mm GL column (GE Healthcare, USA) at ambient temperature. Isocratic elution was performed with Dulbecco's PBS as mobile phase and a flow rate of 0.15 ml/min. Data were analyzed using Astra 6 software (Wyatt Technology Corporation, USA) using a refractive index increment value of 0.185 ml/g.

### PNGase F enzymatic glycan release followed by MALDI-TOF mass spectrometry

To 100 μg of CSL040 was added 20 units of PNGase F (Sigma–Aldrich, USA) dissolved in 50 mM ammonium bicarbonate, bringing the reaction mixture to pH 8. An equivalent control was prepared, substituting PNGase F with MilliQ water. Samples were incubated at 37 °C for 18 h. The addition of 20 units of PNGase F and incubation steps were repeated a further 2 times to ensure that end point deglycosylation of the protein backbone was achieved. For MALDI matrix preparation, 2,5-dihydroxyacetophenone (DHAP) matrix was produced through addition of 7.6 mg of DHAP dissolved in 375 μl ethanol to 125 μl of 18 mg ml^−1^ diammonium citrate dissolved in distilled water. To 2 μl of each sample was added 2 μl of 2% trifluoroacetic acid and mixed in 1.5 ml Eppendorf tubes. To each of these acidified samples (4 μl) was added 2 μl of 2,5-DHAP matrix. Following 30 cycles of aspiration and dispensing in a 10 μl pipette tip to mix, 0.5 μl of each analyte/matrix mixture was applied to individual sample spots upon a Bruker Anchorchip MALDI target. Spots were allowed to completely dry and crystallize at RT. Human serum albumin, used as calibration standard, mixed with 2,5-DHAP matrix was acidified and mixed with matrix in an identical manner to the samples and applied to target spots adjacent to sample spots. Mass measurements of native and deglycosylated samples of CSL040 were acquired using a Bruker Ultraflextreme MALDI-TOF/TOF instrument (Bruker Daltonik, Germany). Mass spectrometer was operated in linear positive ion mode and with a mass range of *m/z* 15,000 to 200,000 Da. MALDI-TOF spectra for analytes were averaged over 5000 shots, during which laser power was manually adjusted over the range 80 to 100%. Signals corresponding to singly, doubly, triply, and quadruple-charged human serum albumin were used as calibrants for the acquisition method as well as for postacquisition adjustment of sample spectrum calibrations. Mass analysis of the acquired MALDI spectrum was performed using Bruker Compass DataAnalysis software version 4.1 (Bruker Daltonik, Germany), spectra were Gaussian smoothed (5.0 points, 10 cycles), and analyte masses were determined using the signal corresponding with triply charged form to derive intact masses of deglycosylated CSL040 and full width at half maximum mass ranges of N-glycosylated CSL040.

## Differential scanning fluorimetry

About 5 μl of 4× buffer concentrate were dispensed in duplicate in a 384-well plate using the Perkin Elmer Janus liquid handling unit (Perkin Elmer, USA). Proteins to be analyzed were diluted to 0.13 mg/ml in PBS and then spiked with a 1/20 dye stock made up in water to give a 1/400 final dilution in each assay reaction. About 15 μl of protein/dye mixture was dispensed as shown previously into each well of the 384-well plate containing the buffer concentrate. The plate was sealed with optical adhesive cover and centrifuged for 1 min at 3220*g* prior to using the QuantStudio Real-Time PCR instrument (Applied Biosystems, USA). A melt curve was generated by cooling down and holding the temperature for 1 min at 20.0 °C, before ramping up from 20 to 99 °C at a rate of 0.05 °C/s. Protein Thermal Shift software (Applied Biosystems, USA) was used to calculate the transition midpoint (*T*_m_) values from each melting curve using the first derivative function. Contour plots were generated using an in-house application developed to graphically display changes in *T*_m_ values in relation to NaCl concentration (*x*-axis) and pH (*y*-axis).

### PNGase F enzymatic glycan release and 2-aminobenzamide labeling

To 100 μg CSL040 or HuCR1(1971) was added 1 M ammonium bicarbonate with pH 8.6 to a final concentration of 100 mM ammonium bicarbonate. HuCR1 was then reduced using dithiothreitol at 10 mM for 30 min at 65 °C, and then alkylated with iodoacetamide at room temperature for 30 min and buffer exchanged into 50 mM ammonium bicarbonate with pH 8.6. To this was added 40 units of N-Glycosidase F (Roche, Switzerland), and the sample was incubated at 37 °C for 18 h. The N-Glycosidase F treated sample was centrifuged through a 50 kDa Amicon Ultra 4 ultrafilter (Millipore, USA), and the filtrate containing the released N-glycans dried using a GeneVac centrifugal vacuum concentrator. About 23 mg of 2-aminobenzamide (2-AB; Sigma–Aldrich, Australia) labeling reagent was dissolved in 350 μl of dimethyl sulfoxide, then 150 μl glacial acetic acid, with the resulting solution added to 32 mg of sodium cyanoborohydride and mixed thoroughly. About 50 μl of the 2-AB reagent was added to the dried sample and incubated in the dark at 65 °C for 3.5 h. Labeled samples were diluted by adding 1.95 ml of 95% v/v acetonitrile, immediately loaded onto the sample Waters Oasis hydrophilic-lipophilic-balanced 3 cc 60 mg solid-phase extraction cartridge and allowed to drain under gravity. Samples were washed with 95% v/v acetonitrile and then eluted with 35% v/v acetonitrile. The eluate was dried in GeneVac centrifugal vacuum concentrator, and then reconstituted with 20 μl of Milli Q water. After dissolution, 30 μl of acetonitrile was added, and the entire sample was transferred to a HPLC vial for analysis.

### 2-AB glycan analysis

HPLC was performed on a Thermo Dionex Ultimate 3000 system (Thermo Fisher Scientific, USA). The separation of 2-AB glycan derivatives was achieved using a Dionex GlycanPac AXH-1, 1.9 μm, 2.1 × 150 mm column (Thermo Fisher Scientific, USA). Mobile phase A consisted of 100% acetonitrile, and mobile phase B consisted of 50 mM ammonium formate with pH 4.0. The column was maintained at 50 °C at a flow rate of 0.200 ml/min. The column was equilibrated with 10% B. After injection of 6 μl of sample, the mobile phase composition was changed linearly to 40% B over 75 min, then 95% B over 10 min, then 95% B for 10 min, and then back to 10% B for 0.1 min. Detection was by fluorescence using an excitation wavelength of 250 nm and an emission wavelength of 420 nm.

### Pharmacokinetic studies

CSL040 and HuCR1(1971) were injected into mixed sex and age wildtype mice (Animal Resources Centre, Australia) as an i.v. bolus at 30 and 43.2 mg/kg, respectively (to reflect a 1:1 M ratio). Time points used for plasma sampling were at 5 min, 0.5 h, 1 h, 2 h, 4 h, 8 h, 16 h, and 48 h. Three mice were used to sample each time point. A staggered sampling strategy was used such that each mouse was bled at an early time point by submandibular bleed and followed by a terminal cardiac bleed. Plasma was collected by puncture of the submandibular vein and for the second plasma sample, by terminal cardiac puncture. Blood was mixed with citrate buffer at a ratio of eight parts blood two parts citrate buffer. Mouse studies were approved by the Institutional Animal Care and Use Committee (Australia).

### HuCR1 concentrations

HuCR1 concentrations were measured by sandwich ELISA in a microtiter plate format using two mouse mAbs specific for HuCR1. Microtiter plates were coated with an anti-CD35 mouse IgG1 monoclonal antibody (J3D3; Beckman Coulter), blocked with 1% casein blocker solution, then diluted rat plasma samples, positive controls, and standards added. Serum levels of HuCR1 test articles were subsequently detected using a biotinylated anti-CD35 mouse IgG1 monoclonal antibody (To5; Thermo Scientific). Horseradish peroxidase (HRP) enzyme was conjugated to the bound biotinylated anti-CD35 antibody by addition of streptavidin-HRP. Following HRP conjugation, 3,3′5,5′-tetramethylbenzidine (TMB) and hydrogen peroxide were added to initiate HRP-mediated oxidation of TMB to generate TMB diimine, a blue color compound that turns to yellow when the reaction is terminated by addition of 0.5 M sulfuric acid. Absorbance at 450 nm was measured using an M200Pro plate reader (Tecan Group Ltd, Switzerland), with the absorbance directly proportional to the amount of HuCR1 test article present in the test samples.

### Pharmacokinetic analysis

The pharmacokinetic analysis was conducted by heuristic noncompartmental methods. Any values below the lower limit of quantitation were not included in the pharmacokinetic analysis. The measured plasma concentration value measured at time *t* is denoted by Y*t*. Tfirst and Tlast denote the time of the first and last positive value of Y*t*, respectively, and Yfirst and Ylast the corresponding plasma concentration at Tfirst and Tlast, respectively. Y0, the plasma concentration immediately after administration, was calculated as the value obtained by linearly extrapolating the first two observed values Y*t* back to zero. If the first value was lower than the second value, the first value was used as Y0. The maximum extrapolated plasma level Cmax was defined as Y0. *In vivo* recovery was defined as Cmax divided by dose multiplied by plasma volume. For simplicity, plasma volume was assumed to be 40 ml/kg, so that an *in vivo* recovery of 100% corresponds to an initial volume of distribution of 40 ml/kg. The terminal elimination rate constant ʎ was estimated by fitting a linear regression line to the last 3, 4, and 5 ln-transformed plasma levels by ordinary least squares. The model with the largest adjusted R2 value was used for estimating the terminal elimination rate ʎ, the corresponding intercept b, and the modeled plasma level at the last measured time point Plast. AUClast, the area under plasma concentration time curve from (0, Y0) to (Tlast, Ylast), was calculated by the trapezoidal method. The area from zero to infinity (AUCinf) was estimated as AUClast plus Plast/ʎ. The fraction of AUCinf that was obtained by extrapolation (%extr) was calculated as one minus AUClast divided by AUCinf. The area under the first moment curve from (0, Y0) to (Tlast, Ylast) was estimated as the area under the approximated first moment curve, *i.e.*, the interpolated curve multiplied by time *t*. The area under the first moment curve from time zero to infinity (AUMCinf) was estimated by adding (Tlast ∗ Plast/ʎ + Plast/ʎ^2^) for the extrapolation to infinity. Clearance was calculated as dose divided by AUCinf, and MRT was calculated as AUMCinf divided by AUCinf. Initial volume of distribution was calculated as dose divided by Cmax, steady state volume of distribution as clearance times MRT, and terminal volume of distribution as clearance divided by terminal elimination rate.

### Generation of rabbit antimouse GBM antibodies

All animal studies were approved by the Institutional Animal Care and Use Committee, Australia. Rabbit antimouse GBM antiserum was raised against the mouse GBM fraction. Briefly, C57BL/6 mice were euthanized and perfused with 30 ml per mouse of PBS containing 1.25% (w/v) iron oxide (Fe_3_O4, <5 μm; Sigma–Aldrich, Australia) into the left ventricle after lancing the vena cava caudalis. After completion of the perfusion, kidneys were harvested and minced into 1 to 2 mm^3^ pieces. Kidney samples were then digested at 37 °C for 30 min in Hank's balanced salt solution (Sigma–Aldrich, Australia) containing 2 mg/ml collagenase type I (Worthington Biochemical, USA) and 0.2 mg/ml DNase I (Sigma–Aldrich, Australia). Digested tissue was passed through 100 μm cell strainer on ice, then though a cell separation magnet (DynaMag-15 Magnet; Thermo Fisher Scientific, USA) and washed for about 5 times to isolate glomeruli fractions. The remaining fraction was washed carefully until a pure fraction of 90 to 95% glomeruli was obtained upon microscopic observation. New Zealand white rabbits were immunized subcutaneously with this glomeruli-rich fraction in CFA (Sigma–Aldrich, USA), which was followed by two boosts in incomplete Freund's adjuvant (Sigma–Aldrich, USA) at 4 week intervals. About 90 days after the first immunization, the rabbits were bled, and the immunoreactivity of the antiserum to mouse GBM *in vitro* was assessed by indirect immunofluorescence staining using frozen sections of normal murine kidneys. Rabbit immune serum was heat inactivated at 56 °C for 30 min, and the IgG fraction was purified using a protein G-sepharose column (GE Healthcare Bio-Sciences, Sweden).

### Induction of anti-GBM GN

Anti-GBM GN was induced in 8- to 9-week-old female C57BL/6 mice (Animal Resources Centre, Australia), as described previously with minor modifications (90). Briefly, the mice were intravenously (tail vein) injected on day 0 with rabbit antimouse GBM antibodies in PBS at a subnephritogenic dose of 1.0 mg, which is below the threshold to induce measurable proteinuria in mice. At day 6, the mice were injected with 2.0 mg of MsαRb antibody (HP6017, IgG2a isotype; American Type Culture Collection, USA) i.p. After the MsαRb antibody injection, mice were placed individually in metabolic cages (Tecniplast, Italy) to collect 24-h urine. Urine albumin levels were measured by an ELISA kit (Bethyl Laboratories, USA), and albuminuria per mouse was measured as microgram albumin per 24 h. To determine the effect of CSL040 on anti-GBM–induced GN, mice were i.p. administered with PBS or CSL040 at the indicated doses, 1 h prior to the injection of MsαRb IgG. For histological analyses, mice were sacrificed at the indicated time points, and cryostat kidney sections were assessed for deposition of complement component and IgG and infiltration of neutrophils. Neutrophils were visualized using a rat antimouse Ly6G (Abcam, UK), followed by incubation with goat antirat IgG, coupled to Alexa fluor 488 (Jackson Immunoresearch, USA). Slides were analyzed using a Nikon eclipse 80i fluorescence microscope (Nikon, Japan). Quantification of fluorescence intensity as raw integrated density was performed using ImageJ software. Plasma levels of complement activation fragments C3b/C3c/iC3b were determined by a mouse C3b ELISA kit (Hycult Biotech, the Netherlands) according to the manufacturer's instructions.

### Flow cytometry analysis of kidney-infiltrating neutrophils

Methods of analyzing kidney neutrophils by flow cytometry have been previously described (92). In this study, 3 h after the injection of MsαRb IgG, mice were euthanized and perfused with 60 ml of cold PBS over 5 to 10 min through the left ventricle. Excised kidneys were minced and digested at 37 °C for 30 min in Hank's balanced salt solution (Sigma–Aldrich, Australia) containing 2 mg/ml collagenase type I (Worthington Biochemical, USA) and 0.2 mg/ml DNase I (Sigma Aldrich, Australia). The digests were passed through a 100 μm cell strainer (BD Biosciences, Australia), washed, and resuspended in PBS plus 2% fetal calf serum for flow cytometry. After blocking nonspecific Fc binding with antimouse CD16/32 (93; eBiosciences, Australia), cells were stained with the following mAbs: FITC-conjugated antimouse CD45 (30-F11; BioLegend, USA), Pacific blue-conjugated anti-CD11b (M1/70; eBiosciences, Australia), BV650-conjugated anti-Ly6G (1A8; BioLegend, USA). Matched isotype controls were used to assess the level of nonspecific binding. Data were collected on an LSR Fortessa flow cytometer (BD Biosciences, USA), and postcollection data were analyzed using FlowJo software (version 10; Tree Star). Cells were pregated on live CD45^+^ single cells. Neutrophils were identified as live CD45^+^CD11b^+^Ly6G^+^ cells. Sphero Blank Calibration Beads (BD Biosciences, USA) were used for enumerating absolute cell numbers.

## Data availability

All data are available on request from the authors. Authors confirm that all data are included in the article.

## Conflict of interest

S. W., H. C., A. B. M., T. R., and M. P. H. are listed as inventors on International Patent Publication number WO2019/218009. All authors with the exception of A. G. N., G. A. P., G. M.- R., M. M., and M. S. are CSL shareholders.
