# Supplementary material for: A novel soluble complement receptor 1 fragment with enhanced therapeutic potential
Source: J Biol Chem. 2020 Dec 23;296:100200. doi: 10.1074/jbc.RA120.016127 (PMC7948397; doi:10.1074/jbc.RA120.016127)
Supplement: Figures S1–S9 [file mmc1.pdf]

A novel soluble complement receptor 1 fragment with enhanced therapeutic potential

Sandra Wymann[1], Yun Dai[2], Anup G. Nair[2], Helen Cao[2], Glenn A. Powers[2], Anna Schnell[1], Genevieve Martin-Roussety[2], David Leong[2], Jason Simmonds[2], Kim G. Lieu[2], Mitchell J. de Souza[2], Marcel Mischnik[3], Shirley Taylor[2], Saw Yen Ow[2], Martin Spycher[1], Rebecca E. Butcher[2], Martin Pearse[2], Adrian W. Zuercher[1], Adriana Baz Morelli[2], Con Panousis[2], Michael J. Wilson[2], Tony Rowe[2], Matthew P. Hardy[2*]

Figure S1. SDS-PAGE analysis of transiently expressed recombinant soluble HuCR1 fragments

Figure S2. Effect of a C-terminal 8His-tag on CSL040 potency *in vitro*

Figure S3. SDS-PAGE analysis of plasma-derived C3b and C4b

Figure S4. Inhibition of Factor I mediated C3b cleavage by CSL040 and HuCR1(1971)

Figure S5. Effect of sialylation on CSL040 potency *in vitro*

Figure S6. Pharmacokinetic comparison of CSL040 with low and high asialo content *in vivo*

Figure S7. Induction of anti-GBM Glomerulonephritis

Figure S8. Anti-GBM glomerulonephritis does not result in systemic consumption of plasma C3

Figure S9. Determination of plasma levels of complement activation fragment C3b/C3c/iC3b in anti-GBM glomerulonephritis mouse model

Figure S1

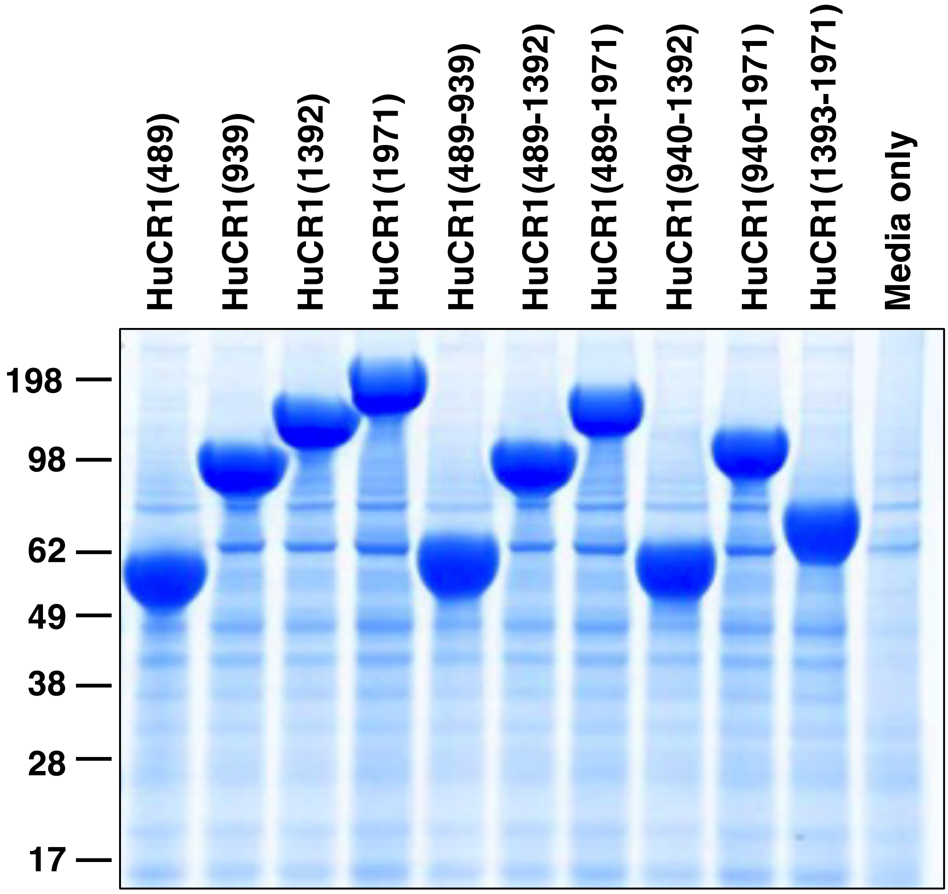

Figure S1. SDS-PAGE analysis of transiently expressed recombinant soluble HuCR1 fragments. Following transient expression of individual recombinant HuCR1 fragments from Expi293F^{TM} cells, culture supernatants were analyzed by SDS-PAGE under non-reducing conditions followed by Coomassie blue staining. The position and size (in kilodaltons) of MW markers are indicated to the left of the gel and above each lane is indicated which supernatant containing HuCR1 fragment was electrophoresed. All soluble recombinant HuCR1 fragments are monomeric under non-reducing conditions in this representative gel.

Figure S2

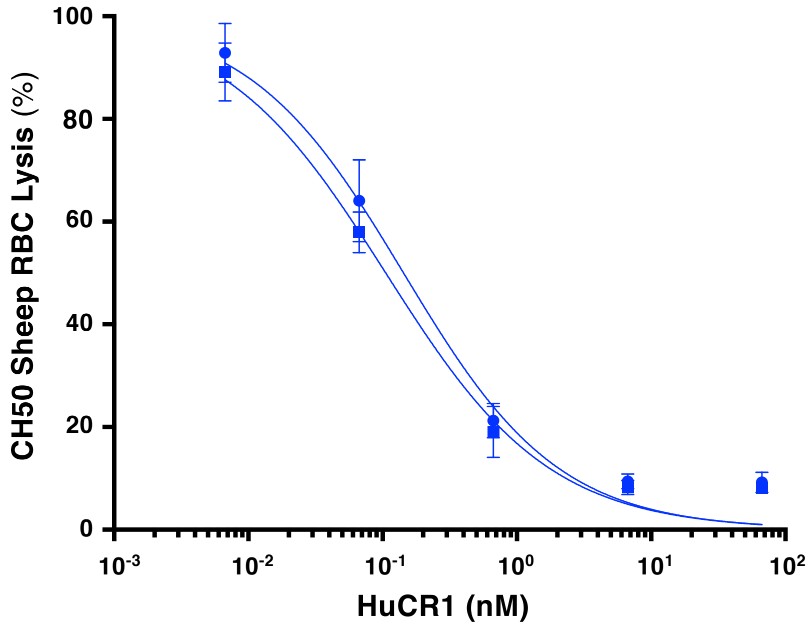

Figure S2. Effect of a C-terminal 8His-tag on CSL040 potency *in vitro*. Classical pathway specific red blood cell hemolytic assays were used to compare the *in vitro* potencies of untagged CSL040 (blue squares) and 8His-tagged CSL040 (blue circles) at a range of doses. Results are expressed as mean ± SD for each concentration, based on N=3 experiments.

Figure S3

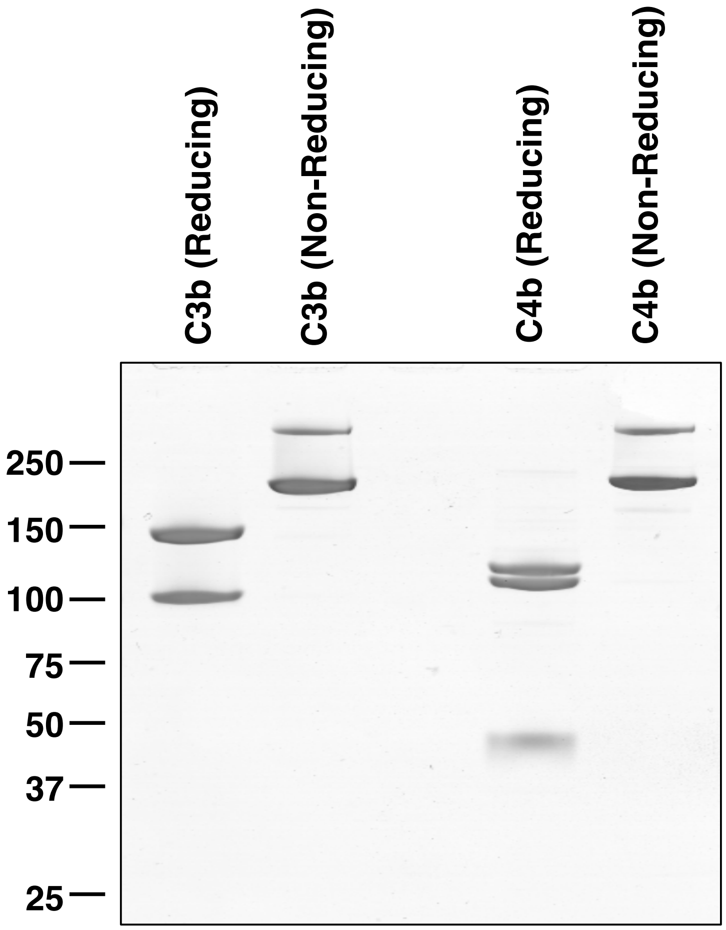

Figure S3. SDS-PAGE analysis of plasma-derived C3b and C4b. 2.5 μg each of Plasma-derived human C3b and C4b sourced from Complement Technology, USA, were analysed by SDS-PAGE under both reducing and non-reducing conditions, followed by Coomassie blue staining. The position and size (in kilodaltons) of MW markers are indicated to the left of this representative gel. Under reducing conditions, C3b is present as alpha and beta chains, and C4b as alpha, beta and gamma chains. Under non-reducing conditions, both C3b and C4b are predominantly monomeric with some dimer.

Figure S4

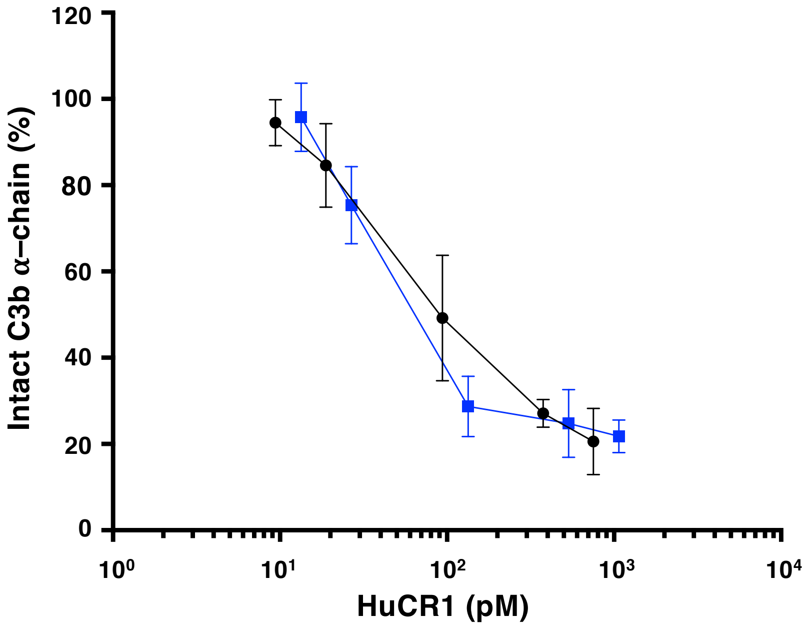

Figure S4. Inhibition of Factor I mediated C3b cleavage by CSL040 and HuCR1(1971). 3 µg C3b and 60 ng Factor I were incubated with increasing concentrations of either CSL040 (blue squares) or HuCR1(1971) (black circles) to induce cleavage of C3b. After incubation, samples were analyzed by SDS-PAGE to separate C3b α-chain from its fragments, followed by Coomassie staining to visualize cleaved and un-cleaved fragments.. Intact C3 α-chain as a percentage of un-cleaved C3b (incubated with Factor I only; set as 100%) was quantified by densitometry and the results are expressed as mean ± SD for each concentration, based on N=3 experiments.

Figure S5

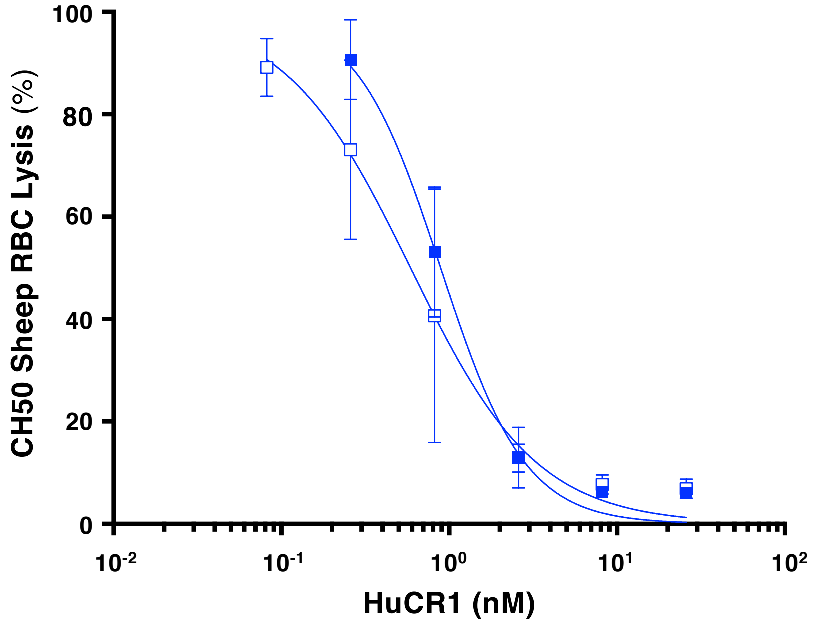

Figure S5. Effect of sialylation on CSL040 potency *in vitro*. Classical pathway specific RBC hemolytic assays were used to compare the *in vitro* potencies of CSL040 containing either high asialo content (filled blue squares) or low asialo content (open blue squares) at a range of doses. Results are expressed as mean ± SD for each concentration, based on N=6 experiments.

Figure S6

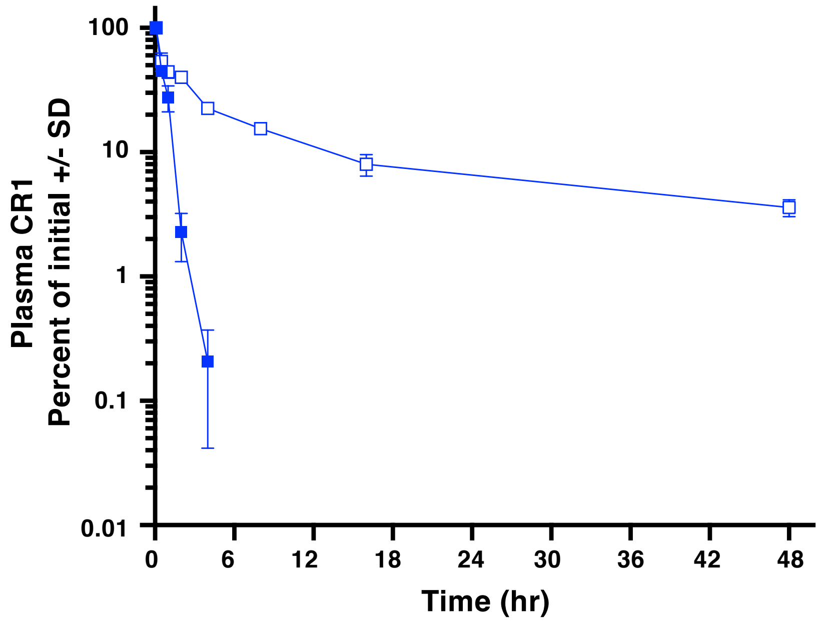

Figure S6. Pharmacokinetic comparison of CSL040 with low and high asialo content *in vivo*. Wild-type mice were administered 30 mg/kg CSL040 as an i.v. bolus, and CSL040 concentrations from 3 serum samples per set time point measured by a CR1-specific ELISA and plotted as a percentage of the first (5 min) dose. The recombinant protein used was generated with either high asialo content (filled squares) or low asialo content (open squares). See Table III for N-glycan content of each test article.

Figure S7

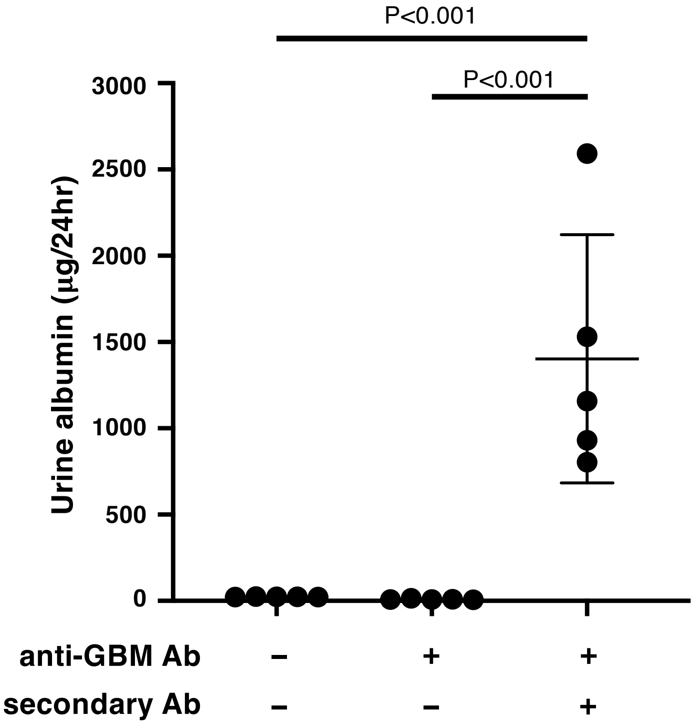

Figure S7. Induction of anti-GBM Glomerulonephritis. C57BL/6 mice were. injected i.v. with either rabbit anti-GBM antibody (1.0 mg in PBS) or PBS alone. After 6 days, PBS or 2 mg MsαRb IgG (secondary Ab) was injected i.p. Urine was collected using metabolic cages and mice were sacrificed after 24 hr. Data shown are mean ± SD. Statistically significant differences in values between groups were calculated using Ordinary One-way ANOVA with the Tukey's test for multiple comparisons.

Figure S8

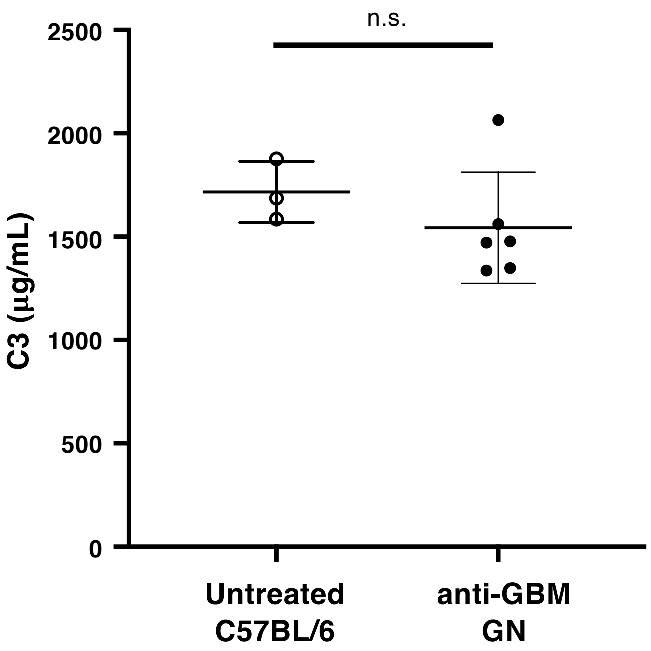

Figure S8. Anti-GBM glomerulonephritis does not result in systemic consumption of plasma C3. Glomerulonephritis (GN) was induced in C57BL/6 mice by i.v. injection of rabbit anti-GBM polyclonal antibody followed 6 days later by i.p. administration of MsαRb antibody. Plasma samples were harvested after 24 hr and C3 levels were measured using an ELISA kit (filled circles) and compared to plasma samples taken from untreated naïve C57BL/6 mice (open circles). Data shown are mean ± SD. Statistically significant differences in values between two groups were calculated using unpaired *t* test; n.s.: not significant.

Figure S9

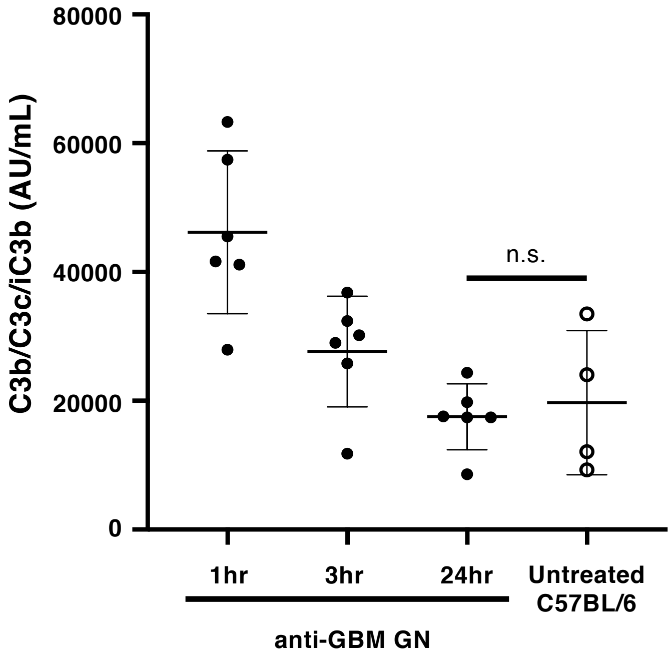

Figure S9. Determination of plasma levels of complement activation fragment C3b/C3c/iC3b in anti-GBM glomerulonephritis mouse model. Glomerulonephritis was induced in C57BL/6 mice by i.v. injection of rabbit anti-GBM polyclonal antibody followed 6 days later by i.p. administration of MsαRb antibody. Plasma was collected 1, 3 and 24 hr after MsαRb administration and C3b/C3c/iC3b levels were measured using an ELISA kit (filled circles) and compared to plasma samples taken from untreated naïve C57BL/6 mice (open circles). Data shown are mean ± SD. No statistically significant difference in values between the 24hr post-Glomerulonephritis induction and the C57BL/6 control groups were observed (Ordinary One-way ANOVA with the Tukey's test for multiple comparisons).
